# Investigations of the Copper Peptide Hepcidin-25 by LC-MS/MS and NMR ^+^

**DOI:** 10.3390/ijms19082271

**Published:** 2018-08-02

**Authors:** Ioana M. Abbas, Marija Vranic, Holger Hoffmann, Ahmed H. El-Khatib, María Montes-Bayón, Heiko M. Möller, Michael G. Weller

**Affiliations:** 1 Federal Institute for Materials Research and Testing (BAM), Division 1.5 Protein Analysis, Richard-Willstätter-Strasse 11, 12489 Berlin, Germany; ioana.abbas@gmail.com (I.M.A.); vranic@uni-potsdam.de (M.V.); 2 School of Analytical Sciences Adlershof, Humboldt-Universität zu Berlin, Unter den Linden 6, 10099 Berlin, Germany; 3 Institute of Chemistry/Analytical Chemistry, University of Potsdam, 14476 Potsdam, Germany; 4 Federal Institute for Materials Research and Testing (BAM), Division 1.8 Environmental Analysis, Richard-Willstätter-Strasse 11, 12489 Berlin, Germany; schach0-0@freenet.de; 5 Department of Chemistry, Humboldt-Universität zu Berlin, Brook-Taylor-Str. 2, 12489 Berlin, Germany; 6 Federal Institute for Materials Research and Testing (BAM), Division 1.1 Inorganic Trace Analysis, Richard-Willstätter-Strasse 11, 12489 Berlin, Germany; ahmed.elkhatib@gmail.com; 7 Department of Pharmaceutical Analytical Chemistry, Faculty of Pharmacy, Ain Shams University, 11566 Cairo, Egypt; 8 Department of Physical and Analytical Chemistry, University of Oviedo, C/Julian Claveria 8, 33006 Oviedo, Spain; montesmaria@uniovi.es

**Keywords:** hepcidin-25, copper, nickel, copper complex, ATCUN motif, metal complex, MS, NMR structure, metal peptide, metalloprotein, metallopeptide, isomerization, racemization, purity, reference material

## Abstract

Hepcidin-25 was identified as the main iron regulator in the human body, and it by binds to the sole iron-exporter ferroportin. Studies showed that the N-terminus of hepcidin is responsible for this interaction, the same N-terminus that encompasses a small copper(II)-binding site known as the ATCUN (amino-terminal Cu(II)- and Ni(II)-binding) motif. Interestingly, this copper-binding property is largely ignored in most papers dealing with hepcidin-25. In this context, detailed investigations of the complex formed between hepcidin-25 and copper could reveal insight into its biological role. The present work focuses on metal-bound hepcidin-25 that can be considered the biologically active form. The first part is devoted to the reversed-phase chromatographic separation of copper-bound and copper-free hepcidin-25 achieved by applying basic mobile phases containing 0.1% ammonia. Further, mass spectrometry (tandem mass spectrometry (MS/MS), high-resolution mass spectrometry (HRMS)) and nuclear magnetic resonance (NMR) spectroscopy were employed to characterize the copper-peptide. Lastly, a three-dimensional (3D) model of hepcidin-25 with bound copper(II) is presented. The identification of metal complexes and potential isoforms and isomers, from which the latter usually are left undetected by mass spectrometry, led to the conclusion that complementary analytical methods are needed to characterize a peptide calibrant or reference material comprehensively. Quantitative nuclear magnetic resonance (qNMR), inductively-coupled plasma mass spectrometry (ICP-MS), ion-mobility spectrometry (IMS) and chiral amino acid analysis (AAA) should be considered among others.

## 1. Introduction

### 1.1. Hepcidin—Bioactivity and Structure

**Figure 1:**
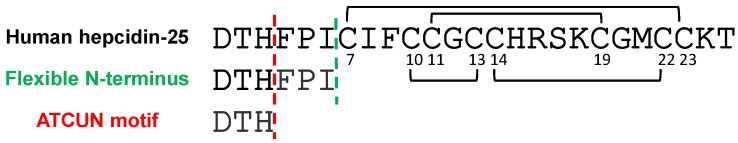
Amino acid sequences of human hepcidin-25 [5], its N-terminus (green) and the ATCUN motif (red).

Hepcidin has attracted much attention ever since its discovery in 2001 [1] as the main player in iron homeostasis. Circulating hepcidin-25 (Hep-25) binds to ferroportin, inducing its internalization and proteolysis and leading to inhibition of intestinal iron absorption [2] (see Supplementary Material Figure S1). Experiments using alanine mutants, in which each residue of the N-terminal region of hepcidin-25 was individually replaced with alanine, established the importance of the structure of the N-terminus for the interaction of Hep-25 with ferroportin [3]. *N*-truncated isoforms (mainly Hep-20, -22, -24) seem to be largely inactive in iron regulation [4]. Intensively discussed work was performed over almost a decade to reveal the solution structure of this peptide hormone. Hep-25 forms a distorted beta sheet stabilized by four intramolecular disulfide bonds [5]. Earlier structural studies of Hep-25 reported the connectivity of the disulfide bonds to be Cys7 to Cys23, Cys10 to Cys22, and Cys11 to Cys19, in addition to a rather unusual vicinal bond between Cys13 and Cys14 [6]. Seven years later, Jordan et al. proved this connectivity to be incorrect and reported the currently accepted disulfide pairing for human hepcidin-25 as follows: Cys7 and Cys23, Cys11 and Cys19, Cys10 and Cys13, and Cys14 and Cys22 [5], forming a rather compact structure. It is important to note that, some recent publications refer (only) to the older 3D structure illustrating that the community is still not fully aware of the current state of structural knowledge and its relevance. A particularity in the structure of Hep-25 is the presence in the N-terminus of a small metal binding site known as ATCUN (amino-terminal Cu(II)- and Ni(II)-binding), which equips Hep-25 with metal binding capacity [7] (Figure 1). Curiously, the paper from Jordan et al. does not refer to the interaction of Hep-25 to metals. The practical reason why synthetic and natural hepcidin-25 have not been found to contain metals is the low pH needed for extraction and purification, which leads to a quantitative loss of any metals bound to the peptide. However, although metal ions are lost under acidic pH, their presence should not be ignored at physiological or higher pH. The fact that the N-terminus, which is responsible for the bioactivity of Hep-25, contains a metal binding site leads to the assumption that the metal might be involved in the interaction of the peptide hormone with ferroportin. In addition, it was shown that the antimicrobial activity of human Hep-25 was enhanced in presence of copper [8]. A recent study on trout hepcidin-25 showed similar results [9].

### 1.2. ATCUN Motif

The ATCUN motif has been studied for more than 50 years [10,11,12,13]. It is a metal binding site specific for the coordination of Cu^2+^ and Ni^2+^, present at the amino terminus of several naturally occurring proteins [14,15,16]. It consists of four nitrogen atoms in the first three N-terminal amino acids, involving the free α-NH_2_ nitrogen, two following amide nitrogen atoms, and an imidazole nitrogen of a histidine residue in the third position. The nitrogen atoms act as metal ligands, forming a distorted square-planar geometry [10,17]. Several biological functions were reported in peptides and proteins due to the presence of the ATCUN motif, such as anticancer and antimicrobial activity, or Cu chelation and transportation [18]. The most well-known and highly abundant protein containing this metal-binding site is human serum albumin (HSA), with the ATCUN motif being responsible for its role in the transport of metal ions, including copper [12]. HSA accommodates Cu^2+^ in its binding site, but this represents only 15% of the total plasmatic copper. The rest is bound to ceruloplasmin (65%), transcuprein (12%), and other serum components of low molecular weight [19] (see Supplementary Material Figure S1). McMillin et al. reported a “free” extracellular copper concentration of 1–2 µM (“unbound” fraction), that was determined by separating the bound fractions of copper through ultrafiltration, using a molecular weight cutoff of 30 kDa, which eliminates albumin, ceruloplasmin, and other proteins expected to bind copper [20], but includes small ligands such as Hep-25. However, due to above-described limitations to isolating the Hep-25-Cu(II) complex, it is still a matter of discussion as to whether hepcidin-25 (2.8 kDa) does exist in the human body in the copper-bound form or not (see Supplementary Material Figure S1). In humans, the physiological level of this peptide is varying from <0.5 nM to around 15 nM [21].

### 1.3. Analysis of Hepcidin-Metal Complexes

Only recently, the interaction of human Hep-25 with metals was explored by other groups. Initially, Farnaud et al. suggested that linear hepcidin-25 binds iron, using MALDI-MS (matrix-assisted laser desorption/ionization mass spectrometry) analysis [22,23]. Later, Tselepis et al. tested the ability of several transition ions (Cu^2+^, Fe^2+^, Fe^3+^, Zn^2+^, and Ni^2+^) to form complexes with the folded hepcidin-25. Their study employing FTICR-MS (Fourier-transform ion cyclotron resonance mass spectrometry) showed no evidence of complex formation in the case of ferrous and ferric ions, while copper, nickel and zinc ions all bound Hep-25 with a sequence of highest-to-lowest affinity as follows: Cu^2+^ > Ni^2+^ > Zn^2+^ [24]. The dissociation constant of the hepcidin-25-copper(II) complex was reported to be <<10^−6^ M. Kulprachakarn et al. further characterized the affinity of Hep-25 for copper using MALDI-MS, with a reported dissociation constant of 10^−7.7^ M. However, the measurements lacking an internal standard provide a poor peptide quantitation, which leads to a higher uncertainty in affinity determination. Recently, Plonka et al. employed potentiometric titration and ultraviolet-visible light (UV-Vis) spectroscopy, which are widely recognized as the standard procedure for the calculation of stability constants of metal complexes [18,25], and characterized the flexible N-terminal hexapeptide of hepcidin-25 (DTHFPI) as the strongest ATCUN ligand ever reported with a dissociation constant of 10^−14.66^ M, which is even higher than the affinity of albumin for copper (K_D_ = 10^−12^ M) [26]. Moreover, this study showed a rapid transfer of Cu^2+^ from HSA to the 6-residue N-terminus [27]. Such extraordinary affinity of the N-terminus of hepcidin-25 for copper is supported by reports, which showed that the conserved presence of aspartic acid (D) as residue 1 in the ATCUN motif increases the basicity of the nitrogen atoms involved in the metal complex, and thus the copper binding is enhanced [28]. Considering this high affinity and the concentration of Cu^2+^ in blood, it is reasonable to assume that a significant fraction of hepcidin-25 is present in the copper-bound form under physiological conditions. In this regard, electrospray ionization (ESI) tandem mass spectrometry (MS/MS) was also employed to characterize the Hep-25-copper complex. Collision-induced dissociation (CID) applied to the copper complex resulted in the fragment DTH-copper, representing the last three residues of the N-terminus of Hep-25 bound to a copper ion. This indicates the stability of the copper-binding motif that is preserved even during MS fragmentation. Furthermore, the affinity of hepcidin-25 for copper allowed the quantification of the peptide in serum samples using inductively coupled plasma mass spectrometry (ICP-MS) by employing direct biomolecule labeling [29]. Publications regarding N-terminus of Hep-25 reported this motif to be unstructured in contrast to the rest of the molecule which is highly rigid due to four intramolecular disulfide bridges [5,6]. This suggests that the N-terminal fragment could serve as a model for metal binding and structural studies, the results of which should then be transferable to full-length hepcidin-25 (Figure 1). Interestingly, the alignment of hepcidin-25 sequences of various species revealed a highly conserved N-terminus. Not only the ATCUN motif (Asp-Thr-His), but even the 6-residue peptide DTHFPI seems to be preserved among most of the mammals (see Supplementary Material Figure S2), which suggests a specific receptor interaction. Despite several studies stating the high affinity of Hep-25 for copper ions, the structure of the human hepcidin-copper complex was so far not elucidated in detail. In studies for the structural analysis of Hep-25 using NMR spectroscopy and molecular dynamics simulations [5,20], the presence of the copper ions was largely ignored. Interestingly, a 3D model of the copper complex of trout hepcidin-25 was proposed [9], however, based on the outdated structural template [6].

### 1.4. Scope of the Present Work

Increasing evidence indicates that the high affinity of hepcidin-25 for copper(II) contributes to its active form and function. This illustrates the importance of the correct and complete structure when working with a biochemical compound, and the need remains to examine the interaction of human hepcidin further. This could have a meaningful impact on hepcidin-25 assays and contribute to solving the problems associated with hepcidin-25 quantification [30,31]. Therefore, the present study investigated the complex formation of hepcidin-25 with metals based on the hypothesis that the natural, bioactive form of human hepcidin-25 contains a copper(II) ion. LC-MS and NMR analysis are employed to investigate the composition and the 3D structure of hepcidin-25 in the metal-bound form with copper(II) and nickel(II). In addition to the structural characterization of Cu^2+^ binding to Hep-25 or its N-terminal ATCUN peptide fragment, we employed Ni^2+^ that is also bound by Hep-25 with high affinity and serves as an appropriate substitute for Cu^2+^ forming a diamagnetic ATCUN complex amenable to high-resolution structure determination by NMR. The copper(II) and nickel(II) complexes of Hep-25 were investigated by NMR spectroscopy under physiological conditions (pH of 7.4). However, LC separation of the transition metal complexes of hepcidin performed at neutral pH was not successful. Therefore, we have chosen to perform LC-MS at basic pH (≈11) in order to avoid abrogation of the metal binding by standard, acidic conditions. These conditions lead to very high stability of the metal complexes. Additionally, high resolution (HR) MS measurements using FTICR-MS were conducted at a pH of 7.4 with the direct infusion technique. 

## 2. Results

### 2.1. Chromatographic Separation of Hepcidin-25-Copper(II) Complexes

Previous MS studies of the hepcidin-25-copper complex using FTICR or MALDI-MS were performed without separating the copper-bound from the copper-free peptide. This hinders an accurate and sensitive analysis of a mixture of the peptide species, especially in more complex matrices such as biological samples. The LC separation improves detection and reduces ion suppression effect in LC-MS analysis.

We introduced an RP-HPLC (reversed-phase high-performance liquid chromatography) separation step complementary to MS detection for a sensitive and robust analysis of the species resulting from the complexation of Hep-25 with copper. This is based on an LC-MS/MS quantification method developed in our laboratory for the quantification of hepcidin-25 using mobile phases containing 0.1% ammonia (pH 11) [32]. For chromatographic separation of the obtained metal species, a neutral or basic pH of the mobile phases is required for complex stability. Unfortunately, a separation of the peptide species at pH 7.4 could not be achieved. It is known that a good chromatographic resolution is possible when fully ionized or fully non-ionized species are used. Hepcidin-25 is a basic peptide with an isoelectric point of 8.5. Thus, hepcidin will be >99% ionized at a pH value approximately 2 pH units above its isoelectric point (10.5). Such high pH values are usually not suitable for commonly used reversed phase chromatography columns that are stable up to a pH of 8. However, the authors employed a new generation of HPLC columns tolerating elevated pH values and providing high performance under these conditions.

**Figure 2:**
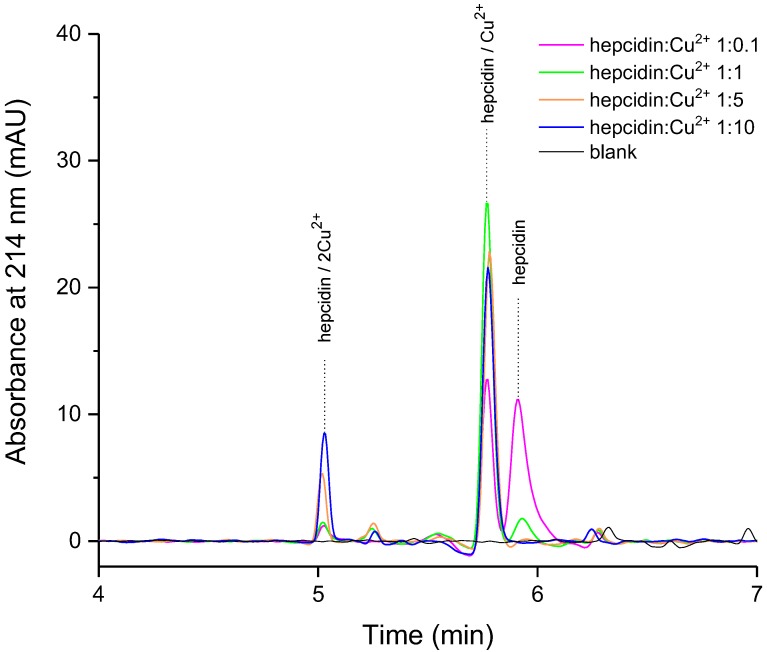
The influence of molar ratio on hepcidin-copper complex behavior (high performance liquid chromatography (HPLC) separation, mobile phase A: H_2_O/NH_3_ 100/0.1 *v*/*v*, pH = 11, B: ACN/H_2_O/NH_3_ 90/10/0.1, concentration of hepcidin-25 of 50 mg/L (18 µM)).

Synthetic hepcidin-25 was titrated with copper ions to monitor the formation of the metal complex(es) by addition of a 1.8–180 µM Cu^2+^ solution to a hepcidin-25 solution of 18 µM (50 mg/L) at a pH of about 11 (0.1% ammonia). Three peaks were identified (Figure 2) that were subsequently attributed via LC-MS analysis to free hepcidin-25, a 1:1 hepcidin-25:copper complex, and the complex of hepcidin-25 with two copper ions.

As expected, the diode array detector (DAD) signal at 214 nm shows a decrease in the apo-hepcidin peak upon the addition of Cu^2+^ due to metal complex formation (Figure 2). Also, the decrease in hepcidin signal at molar ratio 1:5 and 1:10 is accompanied by significantly increased amounts of a new species identified further by MS/MS as the complex of hepcidin with two copper ions. To our best knowledge, this is the first reported chromatographic separation and MS/MS analysis of the peptide complex with two copper ions.

We investigated further the behavior of the metal complex solution prepared at different pH values. At physiological pH (7.4), the species present is mainly the mono-copper complex in accordance with the previously described results at a pH of 6.8 [24] (see Supplementary Material Figure S4). We report the formation of the peptide complex with two copper ions preferably at high pH (11), but not at physiological pH. Further, we characterized the three peaks obtained by tandem mass spectrometry (MS/MS).

### 2.2. MS/MS Characterization of Hepcidin-25 and Hepcidin-25-Copper(II) Species

Hepcidin, hepcidin-Cu^2+^ (1:1) and hepcidin-Cu^2+^ (1:10) were analyzed on a triple quadrupole in positive mode using a 50 mg/L solution (18 µM) in 14% acetonitrile and 0.1% ammonia (starting conditions of the HPLC gradient).

**Figure 3:**
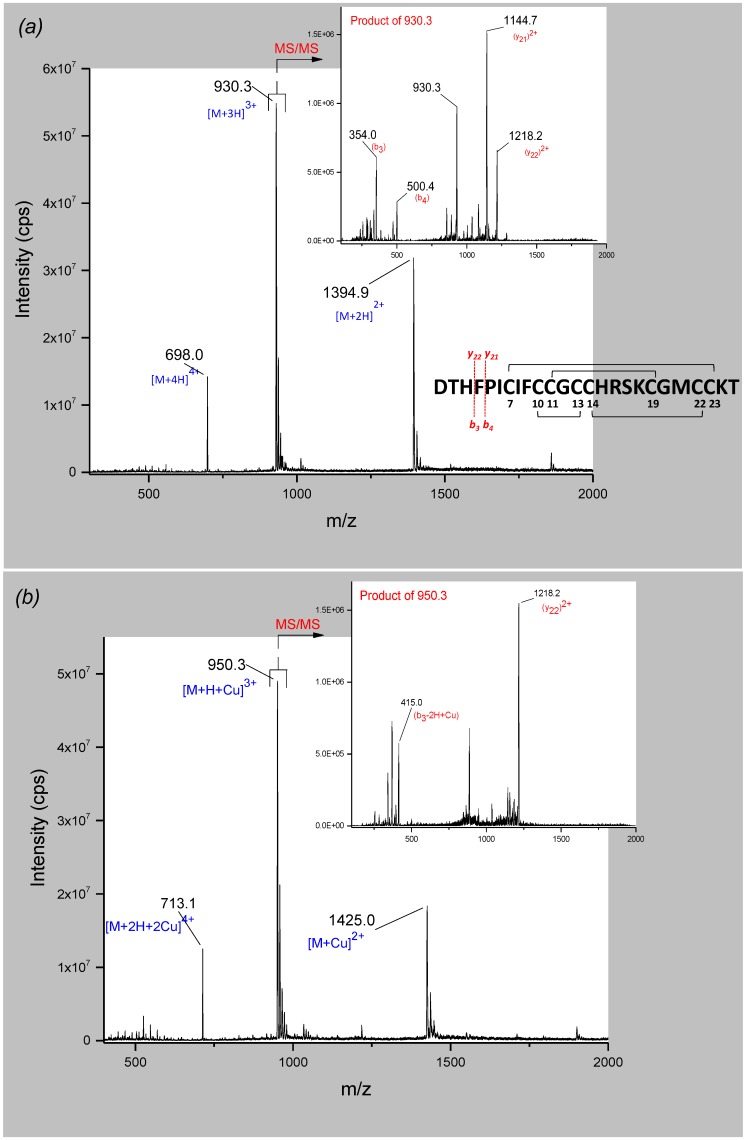
Full scan (mass spectrometry (MS)) and product ion spectra (tandem MS (MS/MS)) of (**a**) hepcidin-25 and (**b**) hepcidin-25-Cu^2+^. The fragmentation pattern presented in (**a**) is valid for both species.

The spectra of hepcidin-25 revealed the doubly, triply, and quadruply charged quasi-molecular ions with *m*/*z* 1394.9 ([M + 2H]^2+^), 930.3 ([M + 3H]^3+^) and 698.0 ([M + 4H]^4+^) respectively. [M + 3H]^3+^ was the most abundant quasi-molecular ion and was selected as precursor ion for fragmentation (Figure 3a). Product ion spectra resulted in the loss of b_3_ (354 Da) to yield y_22_^2+^ (1218.2 Da).

Further, the analysis of the hepcidin-Cu^2+^ solution (molar ratio 1:1) showed a similar series of ions in full scan mode. The quasi-molecular ions with *m*/*z* 1425.0 ([M + C^2+^), 950.3 ([M + H + Cu]^3+^) and 713.1 ([M + 2H + Cu]^4+^) were identified (Figure 3b). The fragmentation of the triply charged quasi-molecular ion as the most intense precursor ion resulted in the same fragment y_22_^2+^ (1218.2 Da) compared to the fragmentation pattern of hepcidin-25, and a fragment of 415 Da which corresponds to a copper-adduct of the N-terminal fragment containing the three amino acids of the ATCUN motif (Asp-Thr-His). The difference of 61 Da corresponds indeed to the addition of one copper (62.9 Da) and the loss of 2 Da and 2 positive charges (2H^+^), in accordance with previous findings [24,29].

Analyzing the solution of hepcidin-Cu^2+^ (molar ratio 1:10) revealed a hepcidin-25 species complexed with two Cu^2+^-ions. The double, triply and quadruply charged quasi-molecular ions were observed at *m*/*z* 1456.1, 971.0, and 728.5, respectively, but the signal intensity was lower compared to the other two species (see Supplementary Material Figure S5). The product ion spectra of the triply charged quasi-molecular ion allowed identification of the 415 Da fragment corresponding to the copper-adduct of the DTH motif. Another fragment of *m*/*z* 1248.7 was identified that could be attributed to a copper-adduct of the fragment (y_22_)^2+^, taking into consideration the mass difference of 30.5 Da for the doubly charged fragment. This indicates a two-Cu^2+^/hepcidin-25 species with one copper ion complexed at the ATCUN motif and a second Cu^2+^ binding at a different site of the peptide. This fact has never been reported before and it can be speculated that the second copper ion binds the histidine residue in position 15, due to the high affinity of the imidazole side chain for copper.

Remarkably, even at pH of 11, the ESI ionization process in positive mode worked efficiently, leading to a sensitive detection of protonated hepcidin species. The negative ion mode was also examined (data not shown) with the triply charged quasi-molecular ion as the most abundant ion, but the sensitivity was poor and we were unable to achieve a specific fragmentation of the precursor ion.

However, the uncertainty of the unit mass resolution provided by the triple quadrupole did not allow accurate determinations of the exact (monoisotopic) mass and the isotope composition. Thus, in order to validate the results obtained with the triple quadrupole and to confirm the isotope pattern of the complexes of hepcidin-25 with copper(II) ions, high-resolution MS (HR-MS) was employed. FTICR-MS investigations showed an isotope pattern for hepcidin-25-copper(II) complexes corresponding to the following chemical formulas: C_113_H_168_N_34_O_31_S_9_Cu and C_113_H_168_N_34_O_31_S_9_Cu_2_ (see Supplementary Material Figure S6). The mass difference between the complex of the peptide with one and two copper ions and hepcidin-25 (chemical formula C_113_H_170_N_34_O_31_S_9_) was 60.9 Da and 123.8 Da respectively (mass accuracy < 4 ppm). This indicates that the interaction with the first copper ion occurs with the loss of two H^+^ as shown previously by LC-MS/MS. The complex of hepcidin-25 with two copper ions is formed at pH 11, with the expulsion of only two protons. At the moment, we have no conclusive explanation for this.

Additionally, we performed FTICR-MS experiment using direct infusion to compare of the behavior of hepcidin-25 complexed with one Cu^2+^ at basic and physiological pH in the absence of the LC mobile phases (pH 11). Very similar isotope patterns of hepcidin-Cu^2+^ at pH values of 7.4 (see Supplementary Material Figure S7) compared to 11 were obtained, indicating no interference of the chromatographic solvents used in the LC-MS/MS analysis on the copper-bound form of the peptide.

FTICR offers superior resolution compared to the triple quadrupole used previously in the LC-MS/MS experiments. However, HR-MS is less attractive for quantification purposes, mainly due to lower sensitivity. In future, the method developed here could represent a valuable tool for the quantification of hepcidin-25/Cu^2+^ in biological samples. However, a prerequisite for this would be the development of a sample preparation protocol at a suitable pH to maintain the copper-bound form of hepcidin-25.

### 2.3. Metal Coordination of Hepcidin-25 and Its N-Terminal Hexapeptide Fragment Monitored by NMR Spectroscopy

With the objective to complement our (LC)-MS studies, the copper(II) and nickel(II) complexes of Hep-25 were investigated by NMR spectroscopy under physiological conditions (pH of 7.4). In order to find out how hepcidin’s ATCUN motif accommodates metal ions at neutral pH, we investigated Cu^2+^ and Ni^2+^ binding of the peptide fragment comprising the six N-terminal amino acids (DTHFPI) and full-length hepcidin-25 by NMR spectroscopy.

First, sequence-specific assignments were achieved by analyzing 1D-^1^H, ^1^H-^13^C-HSQC, 2D- Total Correlation Spectroscopy (TOCSY) and 2D-Rotating Frame Overhauser Enhancement Spectroscopy (ROESY) spectra. In the case of the hexapeptide, these were acquired at room temperature, and a virtually complete assignment was obtained (see Supplementary Material Figure S12). In the case of Hep-25, spectra were acquired at room temperature as well as at 325 K in order to minimize line broadening caused by intermediate dynamics of the loop region [11]. We were able to transfer 84% of the published resonance assignments; however, because of limited sensitivity/peptide concentration in our experiments, the resonance assignment of Hep-25 is still incomplete.

It was possible to detect the proline (Pro-5) *cis*-*trans* isomerization in the hexapeptide and hepcidin-25 by NMR spectroscopy. With respect to the NMR chemical shift timescale, the proline (Pro-5) *cis*/*trans* isomerization is slow, and therefore two sets of resonances are observed in NMR spectra at 298K as well as at 325 K. The *cis* and *trans* isomers in the hexapeptide were distinguished from each other by comparing ^13^C chemical shifts of proline in ^1^H-^13^C HSQC spectra [33] and the *cis*/*trans* ratio is about 30%/70%. It was possible to distinguish the *cis* and *trans* proline isomers in hepcidin-25 by comparison of the TOCSY spectra of hepcidin-25 and hexapeptide (see Supplementary Material Figure S16). As determined from peak intensities, the ratio of the *cis*/*trans* isomers in hepcidin-25 is about 10%/90%, probably caused by the lower flexibility of hepcidin-25 compared to the hexapeptide. Neither the temperature nor the pH value affects the *cis*/*trans* isomer ratio to a significant extent. Proline *cis*-*trans* isomerization is a well-known phenomenon that is frequently observed especially for proline residues in flexible parts of polypeptides. It is of high biological relevance in folding, denaturation, and renaturation of peptides and proteins [34,35].

To characterize metal binding of Cu^2+^ and Ni^2+^, the metals were titrated into solutions of the two peptides and spectral changes were followed by acquiring 1D-^1^H- and 2D-TOCSY spectra. As expected, both peptides showed an affinity for Cu^2+^ as well as Ni^2+^. In case of Cu^2+^, a paramagnetic complex was formed with the ATCUN motif, leading to severe line broadening of residues in close proximity to the coordination site. When Cu^2+^ was added in sub-stoichiometric amounts, two sets of signals arose with one species corresponding to the metal-free peptide showing sharp NMR resonances and one species showing broadened lines of amino acid residues remote from the coordination site and completely vanishing signals of most other residues (see Supplementary Material Figures S9 and S10).

From the TOCSY spectrum taken at an equimolar concentration of Cu^2+^ and hexapeptide (see Supplementary Material Figure S11) it became obvious that only resonances of Ile-6, the residue located furthest away from the Cu^2+^ center, and the signals of Pro-5-Hβ and -Hγ retained detectable intensity. All other signals were completely broadened out. The fact that two sets of signals existed under sub-stoichiometric conditions indicates that the exchange of Cu^2+^ between individual ATCUN motifs is slow on the NMR timescale.

**Figure 4:**
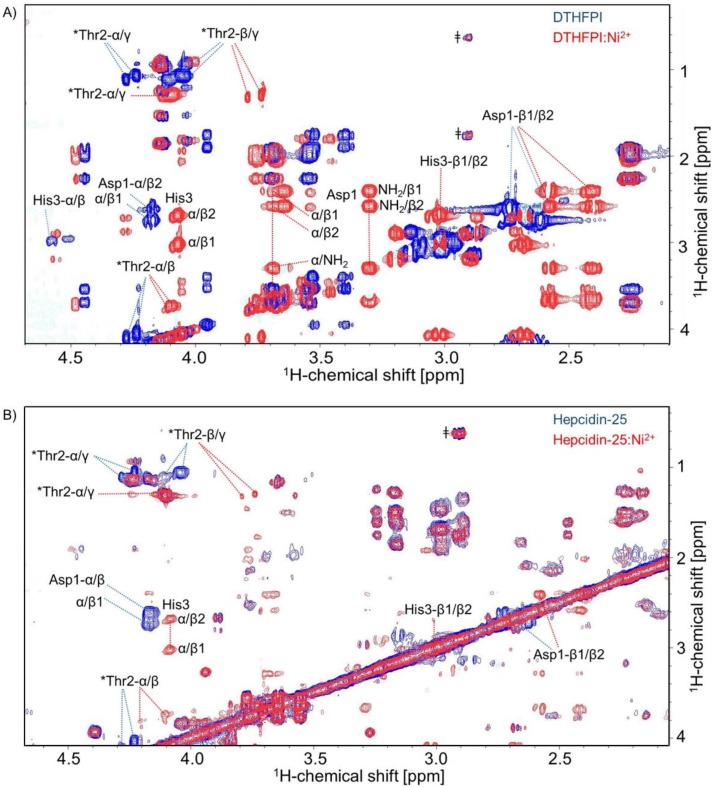
^1^H-^1^H Total Correlation Spectroscopy (TOCSY) spectra of (**A**) hexapeptide DTHFPI and (**B**) hepcidin-25 in the presence of Ni^2+^ (red) and in the absence of Ni^2+^ (blue). The most strongly affected amino acids in both cases are those of the ATCUN motif aspartic acid (Asp-1) (side-chain protons (Hβ)), threonine (Thr2) (alpha proton (Hα) and side-chain protons (Hβ and Hγ) shift) and histidine (His3). * Contains a double set of peaks for each amino acid and suggests the presence of *cis*-trans proline isomerization [11]. ^ǂ^ Indicates the presence of impurities.

As a high-resolution structure determination of Cu^2+^-bound Hep-25 and its N-terminal hexapeptide is impossible because of paramagnetic line broadening, we used Ni^2+^ as a substitute of Cu^2+^. It is known from the literature that both metal ions are bound by ATCUN motifs and show very similar coordination geometries [14,15,16]. Figure 4 illustrates that Ni^2+^ formed a stable complex with hepcidin’s ATCUN motif, both with the DTHFPI hexapeptide (Figure 4A) as well as with full-length Hep-25 (Figure 4B) (See also Supplementary Material Figures S8, S13 and S14), confirming the results by LC-MS shown above. As with Cu^2+^, also the Ni^2+^ complex showed a slow exchange on the NMR timescale.

Coordinating Ni^2+^ causes chemical shift perturbations as a consequence of changes in the electronic structure and by stabilizing a specific conformation. The most strongly affected amino acids were those of the ATCUN motif, specifically aspartic acid (Asp-1), threonine (Thr-2), and histidine (His-3) (Figure 4A).

It is worth noting that upon complexation, the two amino protons of Asp-1 became visible, showing correlations to Asp-1’s α- and β-protons in the TOCSY spectrum. Chemical shift changes due to metal complexation decrease further away from the metal binding site with the smallest effect observed at phenylalanine (Phe-4), proline (Pro-5), and isoleucine (Ile-6). Similar results were obtained for the hepcidin-25-nickel(II) complex by a series of nickel titrations. One-dimensional ^1^H NMR spectra (see Supplementary Material Figure S14) and 2D-TOCSY again revealed the largest chemical shift perturbation at the N-terminal amino acids aspartic acid (Asp-1), threonine (Thr-2) and histidine (His-3) (Figure 4B and Supplementary Material Figure S15). For reasons of sensitivity, we could not exclude small effects on the structure of the C-terminal part of hepcidin-25.

### 2.4. Model-Structure of Hepcidin-25-Copper Complex

Based on the combination of several NMR spectra, structural models of the Cu^2+^-bound hepcidin-25 were constructed. First, the solution structure of the N-terminal hexapeptide complexing Ni^2+^ was determined. Internuclear distance restraints were calculated from ROESY cross peak volumes, and dihedral angle restraints of the peptide backbone were generated from backbone chemical shifts using the program TALOS. In addition, the square-planar coordination known to be present in metal-bound ATCUN motifs was imposed by additional distance and dihedral angle restraints. The resulting 3D structure is shown in the Supplementary Material Figure S17 and the structure determination statistics are summarized in the Supplementary Material Table S1.

Subsequently, models of full-length hepcidin complexing Cu^2+^ were generated by combining the 3D structure of the N-terminal hexapeptide, described above, with the 3D structure of the 19 C-terminal amino acids as determined by Jordan et al. (PDB code: 2KEF) [11].

**Figure 5:**
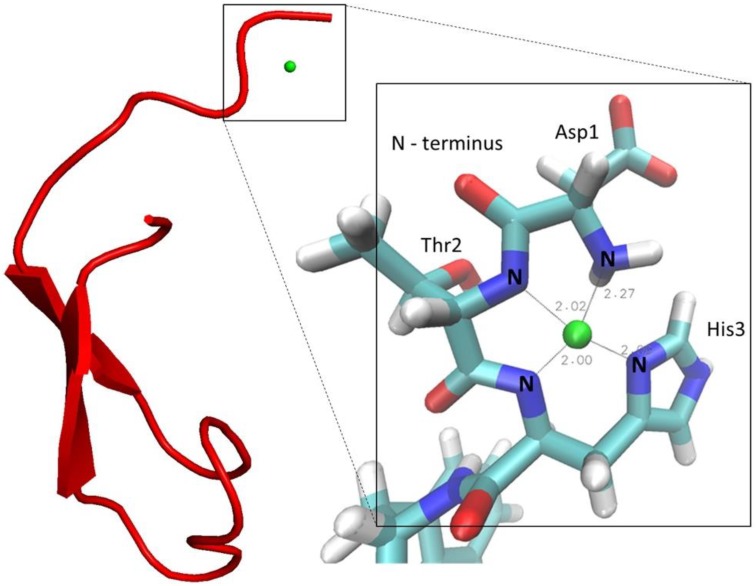
The lowest energy structure of the Cu^2+^-hepcidin-25 model (copper ion in green).

Specifically, we performed simulated annealing calculations with the C-terminus kept fixed by appropriate coordinate restraints. The model has been generated with proline (Pro-5) in the *trans* configuration because this configuration is thermodynamically strongly preferred (~90%). In the N-terminal part, we applied the same distance and dihedral angle restraints that we had previously determined for the Ni^2+^ complex of the hexapeptide. In addition, Ni^2+^ was replaced by Cu^2+^, and the square planar coordination was imposed by restraining distances and dihedral angles as described [13,14,15,16,17,36]. Cu^2+^-N-distances were adjusted to standard values found in X-ray structures of ATCUN motifs [11]. This strategy is justified as it is known that both Ni^2+^ and Cu^2+^ form high-affinity square planar complexes with very similar coordination geometries and metal-ligand bond lengths [11]. The resulting model with the lowest restraint violation energy of copper-bound hepcidin-25 is shown in Figure 5. As a consequence of the coordinate restraints, the C-terminal part corresponds almost perfectly to the 3D structure determined by Jordan et al. [11]. The N-terminus extends away from the disulfide-stabilized C-terminus with some degree of conformational variation as shown in the Supplementary Material Figure S18. This suggests that the linker region connecting the ATCUN motif to the C-terminus is flexible. Laussac et al. suggested that the carboxylate group of the aspartate residue may have some interactions with the metal atoms in the DAH motif, forming a penta-coordinated structure [12]. Currently, we have no evidence in favor of such an additional interaction. However, it cannot be excluded that other residues of hepcidin-25 are involved in weak coordinative interactions to the metal atom in the motif.

## 3. Discussion

The structure and behavior of hepcidin-25 and its metal complexes, particularly of copper(II), were investigated to support the hypothesis that the native form of hepcidin-25 contains a Cu^2+^ ion. In this vein, we present the first LC separation of copper-bound and copper-free hepcidin-25 employing mobile phases that contain 0.1% ammonia (pH 11). In the future, this could lead to the LC-MS/MS quantification of copper-bound hepcidin-25 in biological samples, which might be a superior biomarker in relation to hepcidin-25. At high pH, we identified a new species corresponding to hepcidin-25 complexed with two copper ions. HR-MS experiments showed that two protons are lost during the complex formation of hepcidin-25 with one Cu^2+^, while the second metal ion binds the peptide without H^+^ losses. The first NMR analysis of hepcidin-25 complexed with metal ions was performed. The NMR data recorded for the N-terminus (DTHFPI) complexed with nickel (II) allowed the construction of a 3D model of human hepcidin-25 containing a copper(II) ion coordinated by the ATCUN motif.

By combining LC-MS/MS and NMR spectroscopy, copper (and nickel) complexation by hepcidin-25 could be confirmed and examined in more detail taking advantage of the complementarity of these analytical techniques.

In accordance with previous reports, it could be shown by NMR spectroscopy that Pro-5 of hepcidin undergoes *cis*/*trans* isomerization (about 10%/90%). This is documented by the doubling of NMR resonances observed in the region centered around (Pro-5) [5,6]. It is known that the imide bond of the proline residue can in many instances exist in both the *trans* and the *cis* configuration, in contrast to the peptide bonds involving other amino acid residues, which are rarely found in the *cis* configuration [37]. This *cis*/*trans* isomerization of (Pro-5) in the case of Hep-25 could be of interest for its biological activity and its chromatographic retention properties. Lüders et al. showed that, in the case of salmon antimicrobial peptide (SAMP H1), the isomerization of the proline residue was necessary for the activation of the synthetic peptide. The antimicrobial activity of the synthetic standard was enhanced by treatment with peptidylproline *cis*/*trans*-isomerase [38].

Also, the disulfide connectivity represents another possible cause for hepcidin-25 variation. Ion mobility spectrometry (IMS) studies of two Hep-25 standards from two synthesis pathways showed different mobility profiles. Although the different mobilograms obtained were not further assigned, the authors suggested that multiple distinct conformers were present in these peptide standards [39].

In addition, the isomerization of the aspartate residue (Asp) to iso-aspartate (IsoAsp) is known to occur often, leading to problems such as protein aging or loss of activity [40]. Whether this is relevant in case of the storage of hepcidin should be clarified in future studies. During synthesis and storage, some racemization of amino acids can occur. To our knowledge, none of the materials used for hepcidin analysis have been characterized in this respect, yet. Even simple (non-chiral) amino acid analysis can be very useful for the traceable quantitation or calibration of proteins and particularly peptides [41].

In addition to the formation of metal complexes, several post-translational modifications could contribute to the heterogeneity of Hep-25. These structural modifications may not only affect the biological activity, but also the quantification of the native peptide in biological samples needs to be evaluated accordingly. The current discrepancies between Hep-25 measurements [30] might be caused by the presence of different hepcidin isoforms. Recently, Smith et al. [42] proposed the term “proteoform” to designate all the related molecular forms of a protein product arising from a specific gene. While MS analysis does not easily detect possible isomers, the added value of NMR spectroscopy to the chemical identity allows for high-level purity to be achieved (uncertainties < 0.1% [43]). In this context, quantitative NMR (qNMR) is recommended by the metrological community as a universal and powerful method to be used for purity determination of organic compounds [44,45]. The validity and broader acceptance of qNMR are supported by the use of this methodology by the pharmaceutical and chemical industries in their GMP/GLP settings [46]. Furthermore, the use of ICP-MS could be beneficial for compound-independent calibration based on sulfur or the copper complex [47], and the determination of metal traces [48], particularly copper, potentially introduced from process chemicals and nickel from stainless steel equipment. For the isotopically labeled internal standard, a determination of the isotopic purity would be helpful. Thus, the efforts towards hepcidin-25 standardization [31] could be complemented by the development of a reference material additionally characterized by qNMR, ICP-MS [49] and amino acid analysis [41]. We would like to raise the awareness that synthetic and natural peptides can contain a large number of different proteoforms, from which isomers and metal complexes are particularly difficult to identify. Under different conditions, such as temperature, solvents, pH or salt content, their ratios can be highly variable and might lead to a wrong quantification, even with high-resolution MS. In addition, the use of an insufficiently characterized peptide standard as a calibrator could introduce a hidden bias and might lead to inconsistent results between different laboratories.

## 4. Materials and Methods

### 4.1. Chemicals

Human hepcidin-25 was purchased from Bachem (Bubendorf, Switzerland, H5926.0500, lot number 1060499, >95%) and dissolved in 1% acetic acid (AppliChem, Darmstadt, Germany, 361008.1611, HPLC grade) according to the manufacturer’s instructions. Linear hepcidin-25 was purchased from peptides & elephants (Hennigsdorf, Germany, lot number 1006P06, >95%). Hexapeptide (DTHFPI) was also purchased from peptides & elephants (Hennigsdorf, Germany, >99%). Copper sulfate solution 0.1 M (35185, Titripur^®^) and copper sulfate pentahydrate (C7631) were obtained from Sigma-Aldrich, Taufkirchen, Germany. Nickel sulfate hexahydrate and ammonia solution (30%) (hN66.2, LC-MS grade) were purchased from Carl Roth, Karlsruhe, Germany. Purified laboratory water was obtained from a Milli-Q water-purification system (Millipore, Bedford, MA, USA). Reduced glutathione (Thermo Fischer, Darmstadt, Germany, BP2521-10, >98%) and oxidized glutathione (Sigma-Aldrich, Taufkirchen, Germany, G4376, >98%) were used. Acetonitrile (ACN, 2697, LC-MS grade) was obtained from Chemsolute, Germany and trifluoroacetic acid (TFA, 14264, LC-MS grade) was purchased from Honeywell, Morris Plains, NJ, USA. Sodium dihydrogen phosphate (Carl Roth, Karlsruhe, Germany) and sodium phosphate tribasic 12-hydrate (J. T. Baker^®^, Phillipsburg, NJ, USA) were used. Trimethylsilylpropanoic acid (TSP) was obtained from Sigma-Aldrich, Taufkirchen, Germany. Deuterium oxide (D_2_O) was obtained from Euriso-top (Saint-Aubin, France).

### 4.2. Synthesis of Hepcidin-Metal Complexes by LC-MS

Hepcidin-25 was dissolved in 1% acetic acid solution to obtain a stock solution of 1 mg/mL. Aliquots of 50 µL were stored at −20 °C. The thawed aliquots were used immediately. The synthesis of the Cu^2+^:hepcidin-25 complex was carried out by adding a 1.8–180 µM CuSO_4_ solution in a mixture of the LC mobile phases (defined below) 5% B/95% A (*v*/*v*) to a hepcidin-25 solution of 18 µM (0.1 to 10-fold molar excess). For experiments at different pH values, hepcidin folded in-house was used (see Supplementary Material Figure S3). A solution of 18 µM CuSO_4_ in different buffer solution was added in a molar ratio of 1:1. 100 mM phosphate buffer at a pH of 2.2, 100 mM citrate buffer at a pH of 4.2 and 10 mM ammonium acetate at a pH of 7.4 were used respectively.

### 4.3. LC-MS

LC-MS/MS experiments were carried out on an Agilent 1260 Infinity LC system coupled to a Triple Quad™ 6500 MS (AB Sciex, Darmstadt, Germany). Electrospray ionization was performed in the positive mode (ESI+) with a source temperature of 400 °C. Further parameters used for ionization were 4500 V ion spray voltage, an entrance potential (EP) of 10 V, a curtain gas (CUR) with 35 psi, a nebulizer gas (GS1) with 62 psi, a turbo gas (GS2) with 62 psi and a collision gas (CAD) with 10 psi. Parameters used to produce fragment ions were a declustering potential (DP) of 40 V, a collision cell exit potential (CXP) of 6 V and a collision energy (CE) of 45 V. Data acquisition and analysis were carried out using the software Analyst^®^ 1.6.2 (AB Sciex, Darmstadt, Germany). Chromatographic separation was achieved by an UltraCore 5 μm SuperC18 column (50 mm × 2.1 mm) with an ACE Excel UHPLC pre-column. The mobile phases used for chromatography were composed of 0.1% ammonia in water (pH 11) (A) and in ACN/water, 90/10 (*v*/*v*) (B) respectively. The peptide-copper complexes were eluted applying a flow rate of 600 µL/min as follows: 5% B, isocratic for 2 min, linear increase to 95% B within 5 min, kept at 95% B for 2 min, return to the initial conditions within 0.5 min, and kept for 5.5 min. The column oven temperature was set to 50 °C and the injection volume employed was 10 µL.

### 4.4. FTICR-MS

A Thermo LTQ FTICR Ultra MS was operated in positive mode. Mass calibration was achieved by using a Pierce LTQ ESI Positive Ion Calibration Solution. The system was tuned using a hepcidin-25 solution 5 mg/L dissolved in water/ACN/TFA 60/38/2 v/v/v. Typically, a scanning range of *m*/*z* 400–2000 was detected. Direct infusion was employed using the integrated syringe pump set at a flow rate of 30 µL/min. Hepcidin-25-copper complex solutions at pHs of 11 and 7.4 respectively, prepared as described previously, were analyzed.

### 4.5. NMR Spectroscopy

All NMR experiments were performed on a Bruker AVANCE III 600 spectrometer (Bruker Biospin GmbH, Rheinstetten, Germany) equipped with a 5 mm BBI probe. Chemical shifts were referenced to internal 3-(trimethylsilyl)propanoic-2,2,3,3-d_4_ acid (TSP) at 0.0 ppm. NMR samples were prepared as aqueous solutions containing 5% D_2_O for experiments detecting exchangeable protons. Metal titrations were performed by adding small amounts of concentrated solutions of metal salts to the peptide sample. Ni^2+^ or Cu^2+^ titrations were performed by adding 10 mM NiSO_4_·6H_2_O or CuSO_4_ stock solution, respectively, to the 2.5 mM phosphate-buffered solution of the peptide, at pH of 7.4, until a 1:1 complex was formed. Chemical shift assignments were achieved by analyzing 1D-^1^H, ^1^H-^13^C HSQC, ^1^H, ^1^H-TOCSY, and ^1^H, ^1^H-ROESY spectra. The ROESY mixing time was 400 ms. In case of metal-free hepcidin-25, NMR spectra were recorded at 273 K and 325 K. The spectra of hexapeptide, hexapeptide-metal complex and hepcidin-25-metal complex were acquired at 273 K. In the case of the metal-free peptides, the majority of chemical shift assignments could be transferred from published data [11]. Acquired data were processed and analyzed using Bruker Topspin (v3.5) and the CARA software (v1.9.1) [50].

### 4.6. Molecular Modeling

Structures were calculated with Xplor-NIH v2.43. The model of metal-bound hepcidin-25 was generated by joining the 3D structure of the Ni^2+^-bound hexapeptide that was calculated based on experimental restraints with the C-terminal part of hepcidin-25 with its coordinates fixed to the conformation reported by Jordan et al. [11]. The 3D structure of the Ni^2+^-bound N-terminal hexapeptide (DTHFPI) was obtained by simulated annealing with r^−6^ averaging of distances within Xplor-NIH. Distance restraints were derived from ROESY cross-peak volumes, which were multiplicity corrected and calibrated with reference to cross peak volumes corresponding to known distances in the peptide [51]. For all internuclear distances, we defined upper and lower distance bounds with a large margin (d ± 1 Å) to take into account offset-dependent and other distance uncertainties specific to the ROESY experiment [52,53]. Modifications of the Xplor-NIH parameter and topology files were made to incorporate the nickel or copper ion in a square planar complex, using distance and torsion angle restraints, involving the four nitrogen atoms. The correct square planar geometry of nickel or copper coordination was maintained by imposing 14 restraints including bond distances, bond angles, and dihedral angles. The final ensemble of 10 hexapeptide structures had a backbone r.m.s.d. of 1.17 Å and 75% of backbone dihedral angles were in the most favored region of the Ramachandran plot. Complete structural statistics are provided in the online Supplementary Material (Table S1). Chemical shifts were translated into backbone dihedral angle restraints by using the software TALOS [54]. Calculations were performed according to the standard protocol of Xplor-NIH. The PDB structure of hepcidin-25 (code 2KEF) [11] was used as a starting structure for simulated annealing. Simulated annealing was done with 24,000, 40-psec, steps at 1000 K during heating and 3000, 0.8-psec, steps during cooling to 100 K.

## Supplementary Materials

The following are available online at http://www.mdpi.com/1422-0067/19/8/2271/s1.

## References

[1] Park C.H., Valore E.V., Waring A.J., Ganz T. (2001). Hepcidin, a urinary antimicrobial peptide synthesized in the liver. J. Biol. Chem..

[2] Nemeth E., Tuttle M.S., Powelson J., Vaughn M.B., Donovan A., Ward D.M., Ganz T., Kaplan J. (2004). Hepcidin regulates cellular iron efflux by binding to ferroportin and inducing its internalization. Science.

[3] Clark R.J., Tan C.C., Preza G.C., Nemeth E., Ganz T., Craik D.J. (2011). Understanding the structure/activity relationships of the iron regulatory peptide hepcidin. Chem. Biol..

[4] Laarakkers C.M., Wiegerinck E.T., Klaver S., Kolodziejczyk M., Gille H., Hohlbaum A.M., Tjalsma H., Swinkels D.W. (2013). Improved mass spectrometry assay for plasma hepcidin: Detection and characterization of a novel hepcidin isoform. PLoS ONE.

[5] Jordan J.B., Poppe L., Haniu M., Arvedson T., Syed R., Li V., Kohno H., Kim H., Schnier P.D., Harvey T.S. (2009). Hepcidin revisited, disulfide connectivity, dynamics, and structure. J. Biol. Chem..

[6] Hunter H.N., Fulton D.B., Ganz T., Vogel H.J. (2002). The solution structure of human hepcidin, a peptide hormone with antimicrobial activity that is involved in iron uptake and hereditary hemochromatosis. J. Biol. Chem..

[7] Melino S., Garlando L., Patamia M., Paci M., Petruzzelli R. (2005). A metal-binding site is present in the structure of hepcidin. J. Pept. Res..

[8] Maisetta G., Petruzzelli R., Brancatisano F.L., Esin S., Vitali A., Campa M., Batoni G. (2010). Antimicrobial activity of human hepcidin 20 and 25 against clinically relevant bacterial strains: Effect of copper and acidic pH. Peptides.

[9] Alvarez C.A., Guzman F., Cardenas C., Marshall S.H., Mercado L. (2014). Antimicrobial activity of trout hepcidin. Fish Shellfish Immunol..

[10] Camerman N., Camerman A., Sarkar B. (1976). Molecular design to mimic the copper(II) transport site of human albumin. The crystal and molecular structure of copper(II)—Glycylglycyl-l-histidine-*N*-methyl amide monoaquo complex. Can. J. Chem..

[11] Hartford C., Sarkar B. (1997). Amino Terminal Cu(II)- and Ni(II)-Binding (ATCUN) Motif of Proteins and Peptides Metal Binding, DNA Cleavage, and Other Properties. Acc. Chem. Res..

[12] Laussac J.-P., Sarkar B. (1984). Characterization of the copper(II)- and Ni transport site of HSA. Studies of Cu and Ni binding to peptide 1-24 od HSA by C and H Spectroscopy. Biochemistry.

[13] Sankararamakrishnan R., Verma S., Kumar S. (2005). ATCUN-like metal-binding motifs in proteins: Identification and characterization by crystal structure and sequence analysis. Proteins.

[14] Bal W., Jezowska-Bojczuk M., Kasprzak K.S. (1997). Binding of Nickel(II) and Copper(II) to the *N*-Terminal Sequence of Human Protamine HP2. Chem. Res. Toxicol..

[15] Gasmi G., Singer A., Forman-Kay J., Sarkar B. (1996). NMR structure of neuromedin C, a neurotransmitter with an amino terminal CuII-, NiII-binding (ATCUN) motif. J. Pept. Res..

[16] Grogan J., McKnight C.J., Troxler R.F., Oppenheim F.G. (2001). Zinc and copper bind to unique sites of histatin 5. FEBS Lett..

[17] Hureau C., Eury H., Guillot R., Bijani C., Sayen S., Solari P.L., Guillon E., Faller P., Dorlet P. (2011). X-ray and solution structures of Cu(II) GHK and Cu(II) DAHK complexes: Influence on their redox properties. Chemistry.

[18] Faller P., Gonzalez P., Bossak K., Stefaniak E., Hureau C., Raibaut L., Bal W. (2018). *N*-terminal Cu Binding Motifs Xxx-Zzz-His (ATCUN) and Xxx-His and their derivatives: Chemistry, Biology and Medicinal Applications. Chemistry.

[19] Linder M.C., Hazegh-Azam M. (1996). Copper biochemistry and molecular biology. Am. J. Clin. Nutr..

[20] McMillin G.A., Travis J.J., Hunt J.W. (2009). Direct measurement of free copper in serum or plasma ultrafiltrate. Am. J. Clin. Pathol..

[21] WCX-TOF MS reference values for serum Hepcidin-25. www.hepcidinanalysis.com.

[22] Farnaud S., Patel A., Evans R.W. (2006). Modelling of a metal-containing hepcidin. Biometals.

[23] Farnaud S., Rapisarda C., Bui T., Drake A., Cammack R., Evans R.W. (2008). Identification of an iron-hepcidin complex. Biochem. J..

[24] Tselepis C., Ford S.J., McKie A.T., Vogel W., Zoller H., Simpson R.J., Diaz Castro J., Iqbal T.H., Ward D.G. (2010). Characterization of the transition-metal-binding properties of hepcidin. Biochem. J..

[25] Thordarson P. (2011). Determining association constants from titration experiments in supramolecular chemistry. Chem. Soc. Rev..

[26] Kulprachakarn K., Chen Y.L., Kong X., Arno M.C., Hider R.C., Srichairatanakool S., Bansal S.S. (2016). Copper(II) binding properties of hepcidin. J. Biol. Inorg. Chem..

[27] Płonka D., Bal W. (2017). The *N*-terminus of hepcidin is a strong and potentially biologically relevant Cu(II) chelator. Inorg. Chim. Acta.

[28] Miyamoto T., Kamino S., Odani A., Hiromura M., Enomoto S. (2013). Basicity of *N*-Terminal Amine in ATCUN Peptide Regulates Stability Constant of Albumin-like Cu^2+^ Complex. Chem. Lett..

[29] Konz T., Montes-Bayon M., Sanz-Medel A. (2012). Elemental labeling and isotope dilution analysis for the quantification of the peptide hepcidin-25 in serum samples by HPLC-ICP-MS. Anal. Chem..

[30] Kroot J.J., van Herwaarden A.E., Tjalsma H., Jansen R.T., Hendriks J.C., Swinkels D.W. (2012). Second round robin for plasma hepcidin methods: First steps toward harmonization. Am. J. Hematol..

[31] Van der Vorm L.N., Hendriks J.C., Laarakkers C.M., Klaver S., Armitage A.E., Bamberg A., Geurts-Moespot A.J., Girelli D., Herkert M., Itkonen O. (2016). Toward Worldwide Hepcidin Assay Harmonization: Identification of a Commutable Secondary Reference Material. Clin. Chem..

[32] Abbas I.M., Hoffmann H., Montes-Bayon M., Weller M.G. (2018). Improved LC-MS/MS method for the quantification of hepcidin-25 in clinical samples. Anal. Bioanal. Chem..

[33] Lee Y.-C., Jackson P.L., Jablonsky M.J., Muccio D.D., Pfister R.R., Haddox J.L., Sommers C.I., Anantharamaiah G.M., Chadda M. (2001). NMR conformational analysis of *cis* and *trans* proline isomers in the neutrophil chemoattractant, *N*-acetyl-proline-glycine-proline. Biopolymers.

[34] Andreotti A.H. (2003). Native State Proline Isomerization: An Intrinsic Molecular Switch. Biochemistry.

[35] Brandts J.F., Halvorson H.R., Brennan M. (1975). Consideration of the possibility that the slow step in protein denaturation reactions is due to cis-trans isomerism of proline residues. Biochemistry.

[36] Yu Y., Zhou M., Kirsch F., Xu C., Zhang L., Wang Y., Jiang Z., Wang N., Li J., Eitinger T. (2014). Planar substrate-binding site dictates the specificity of ECF-type nickel/cobalt transporters. Cell Res..

[37] Stewart D.E., Sarkar A., Wampler J.E. (1990). Occurrence and role of *cis* peptide bonds in protein structures. J. Mol. Biol..

[38] Luders T., Birkemo G.A., Nissen-Meyer J., Andersen O., Nes I.F. (2005). Proline conformation-dependent antimicrobial activity of a proline-rich histone h1 *N*-terminal Peptide fragment isolated from the skin mucus of Atlantic salmon. Antimicrob. Agents Chemother..

[39] Bros P., Josephs R.D., Stoppacher N., Cazals G., Lehmann S., Hirtz C., Wielgosz R.I., Delatour V. (2017). Impurity determination for hepcidin by liquid chromatography-high resolution and ion mobility mass spectrometry for the value assignment of candidate primary calibrators. Anal. Bioanal. Chem..

[40] Wakankar A.A., Borchardt R.T., Eigenbrot C., Shia S., Wang Y.J., Shire S.J., Liu J.L. (2007). Aspartate Isomerization in the Complementarity-Determining Regions of Two Closely Related Monoclonal Antibodies. Biochemistry.

[41] Hesse A., Weller M.G. (2016). Protein Quantification by Derivatization-Free High-Performance Liquid Chromatography of Aromatic Amino Acids. J. Amino Acids.

[42] Smith L.M., Kelleher N.L. (2013). The Consortium for Top Down Proteomics. Proteoform: A single term describing protein complexity. Nat. Methods.

[43] Weber M., Hellriegel C., Rück A., Sauermoser R., Wüthrich J. (2013). Using high-performance quantitative NMR (HP-qNMR®) for certifying traceable and highly accurate purity values of organic reference materials with uncertainties <0.1%. Accredit. Qual. Assur..

[44] Huang T., Zhang W., Dai X., Zhang X., Quan C., Li H., Yang Y. (2014). Precise measurement for the purity of amino acid and peptide using quantitative nuclear magnetic resonance. Talanta.

[45] Malz F., Jancke H. (2005). Validation of quantitative NMR. J. Pharm. Biomed. Anal..

[46] Pauli G.F., Chen S.N., Simmler C., Lankin D.C., Godecke T., Jaki B.U., Friesen J.B., McAlpine J.B., Napolitano J.G. (2014). Importance of purity evaluation and the potential of quantitative ^1^H NMR as a purity assay. J. Med. Chem..

[47] Konz T., Montes-Bayón M., Bettmer J., Sanz-Medel A. (2011). Analysis of hepcidin, a key peptide for Fe homeostasis, viasulfur detection by capillary liquid chromatography-inductively coupled plasma mass spectrometry. J. Anal. At. Spectrom..

[48] Vergote V., Burvenich C., Van de Wiele C., De Spiegeleer B. (2009). Quality specifications for peptide drugs: A regulatory-pharmaceutical approach. J. Pept. Sci..

[49] Bernevic B., El-Khatib A.H., Jakubowski N., Weller M.G. (2018). Online immunocapture ICP-MS for the determination of the metalloprotein ceruloplasmin in human serum. BMC Res. Notes.

[50] Keller R. (2004). The Computer Aided Resonance Assignment Tutorial.

[51] Würthrich K. (1986). NMR of Proteins and Nucleic Acids.

[52] Bax A. (1988). Correction of Cross-Peak Intensities in 2D Spin-Locked NOE Spectroscopy for Offset and Hartmann-Hahn Effects. J. Magn. Reson..

[53] Bax A., Davies D.G. (1985). Practical Aspects of Two-Dimensional Transverse NOE Spectroscopy. J. Magn. Reson..

[54] Cornilescu G., Delaglio F., Bax A. (1999). Protein backbone angle restraints from searching a database for chemical shift and sequence homology. J. Biomol. NMR.

