# Supplementary material for: Investigations of the Copper Peptide Hepcidin-25 by LC-MS/MS and NMR +
Source: Int J Mol Sci. 2018 Aug 2;19(8):2271. doi: 10.3390/ijms19082271 (PMC6121404; doi:10.3390/ijms19082271)
Supplement: Supplementary file 1 [file ijms-19-02271-s001.pdf]

Supplementary Material

Investigations of the copper peptide hepcidin-25 by LC-MS/MS and NMR*

Ioana M. Abbas[1,2*], Marija Vranic[2,3*], Holger Hoffmann[4,5], Ahmed H. El-Khatib[6,7], María Montes-Bayón[8], Heiko M. Möller[3+] and Michael G. Weller[1+]

[1] Federal Institute for Materials Research and Testing (BAM), Division 1.5 Protein Analysis, Richard-Willstätter-Strasse 11, 12489 Berlin, Germany
[2] Humboldt-Universität zu Berlin, School of Analytical Sciences Adlershof, Unter den Linden 6, 10099 Berlin, Germany
[3] Institute of Chemistry/Analytical Chemistry, University of Potsdam, 14476 Potsdam, Germany
[4] Federal Institute for Materials Research and Testing (BAM), Division 1.8 Environmental Analysis, Richard-Willstätter-Strasse 11, 12489 Berlin, Germany
[5] Humboldt-Universität zu Berlin, Department of Chemistry, Brook-Taylor-Str. 2, 12489 Berlin, Germany
[6] Federal Institute for Materials Research and Testing (BAM), Division 1.1 Inorganic Trace Analysis, Richard-Willstätter-Strasse 11, 12489 Berlin, Germany
[7] Department of Pharmaceutical Analytical Chemistry, Faculty of Pharmacy, Ain Shams University, Cairo, Egypt
[8] Department of Physical and Analytical Chemistry, University of Oviedo, C/Julian Claveria 8, 33006, Oviedo, Spain

* Both authors contributed equally to this paper.
+ Correspondence:
michael.weller@bam.de; Tel.: +49-30-8104-1150, or
heiko.moeller@uni-potsdam.de, Tel. +49-331-977-5425

* Dedicated to Dr. Norbert Jakubowski on the occasion of his 65th birthday.

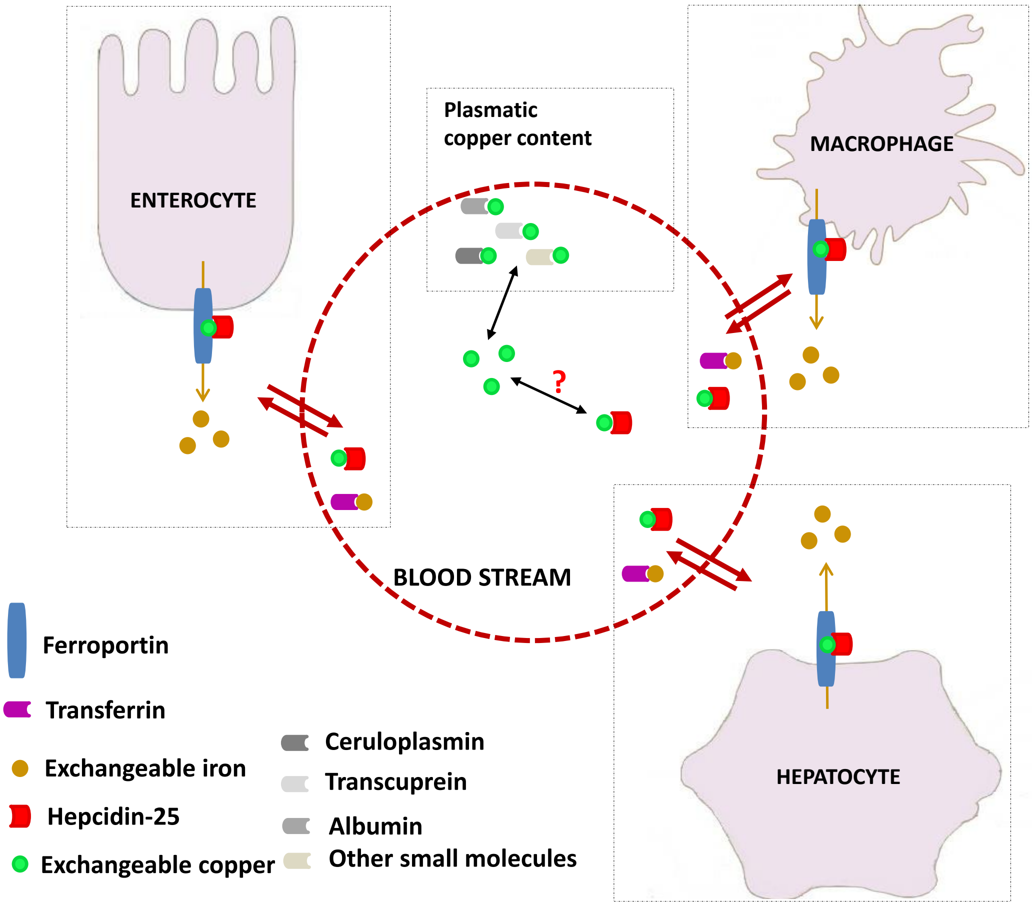

Fig 1 – Iron homeostasis. Suggested iron and copper traffic in the bloodstream. Ferroportin (blue) is expressed on the membrane surface of iron releasing cells (enterocytes, hepatocytes, and macrophages), allowing the iron ions (yellow) to exit the cell (yellow arrow) and bind transferrin (pink) for transport through the blood stream. Circulating hepcidin-25 (red) blocks the iron export by binding to ferroportin. Copper ions (green) released from intestinal cells are bound by various molecules in the blood. Iron and copper ions are exchanged between various complexing agents based on chemical affinities and biological needs.

Salmon (Q801Y3)	QIHLSL CGLCCNCCHNIGCGFCCKF
Mouse (Q9EQ21)	DTNFPI CIFCCKCCNNSQCGICCKT
Chimpanzee (A7XEH6)	DTHFPI CIFCCGCCHRSKCGMCCKT
Pig (Q8MJ80)	DTHFPI CIFCCGCCRKAICGMCCKT
Dog (Q5U9D2)	DTHFPI CIFCCGCCKTPKCGLCCKT
Sheep (E0X9N1)	DTHFPI CIFCCGCCRKGTCGICCKT
Rabbit (G1U3P2)	DTHFPI CIFCCSCCRNSKCGICCKT
Bovine (Q2NKT0)	DTHFPI CIFCCGCCRKGTCGMCCRT
Human (P81172)	DTHFPI CIFCCGCCHRSKCGMCCKT

Fig 2 – Hepcidin-25 sequences of various species. The first 6-residue sequence is delimited (green line). Amino acids which differ from the sequence of human hepcidin-25 are shown in orange.

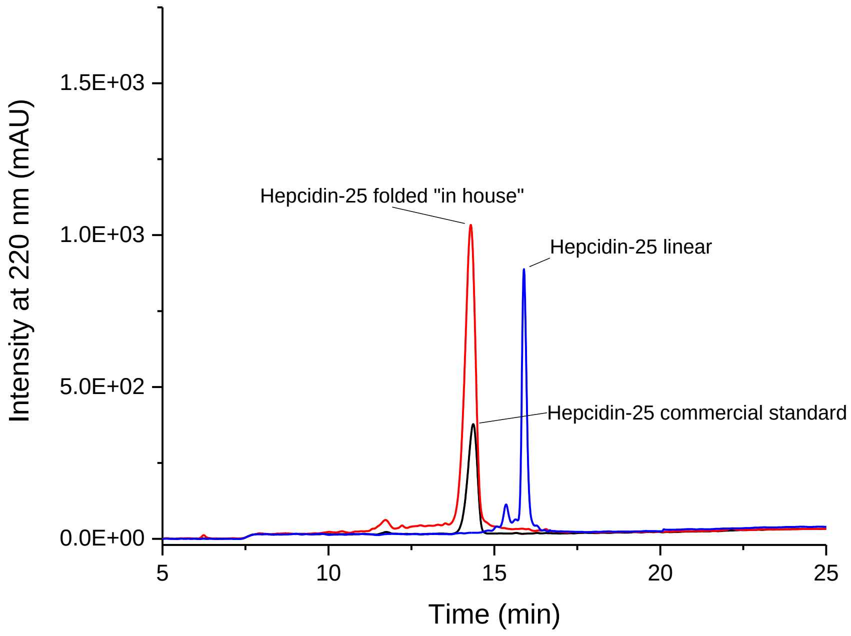

Fig. 3 – HPLC chromatograms of linear and folded hepcidin-25 (linear hepcidin-25 50 µM – blue, folded commercial hepcidin-25 25 µM – black, folded "inhouse" hepcidin-25 50 µM – red)

Folding protocol: *Linear hepcidin-25 was dissolved in water at a concentration of 10 mg/mL. The solution was aliquoted into 100 µL aliquots and immediately stored at -20°C. The aliquots were thawed right before use. 1 mg of linear hepcidin-25 was dissolved in 30 mL folding solution containing acetonitrile and folding buffer 20:80 (v/v). Reduced and oxidized glutathione (GSH and GSSG respectively) were used for the preparation of the folding buffer (GSSG 0.4 mM, GSH 0.4 mM) [10] at a pH of 7. Linear hepcidin-25 was oxidized overnight under stirring. The solution was acidified by addition of TFA (0.1%) and further loaded onto a Zorbax C18 column, 4.6x150mm, 5µm (Agilent Technologies, Ratingen, Germany) for semi-preparative purification. Mobile phases consisting of A: TFA 0.1% in water and B: 0.08% TFA in ACN were used in a linear gradient of 20%B to 60%B in 30 min at a flow rate of 1 mL/min. The column oven was set to 50°C.*

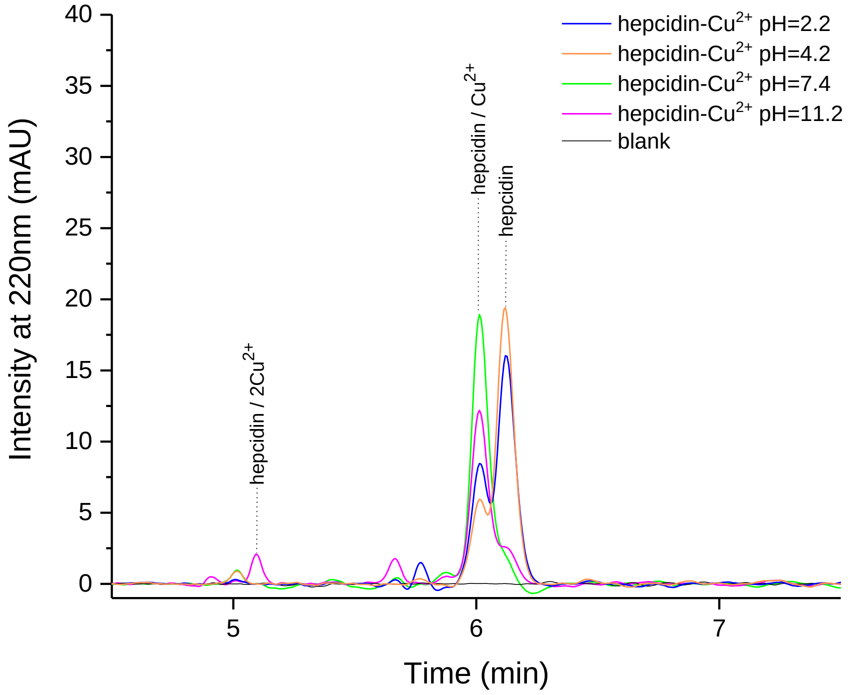

Fig. 4 – The influence of sample pH on hepcidin-25-copper complexes (HPLC separation, mobile phase A: H_2O/NH_3 100/0.1 v/v, pH=11, B: ACN/ H_2O/NH_3 90/10/0.1)

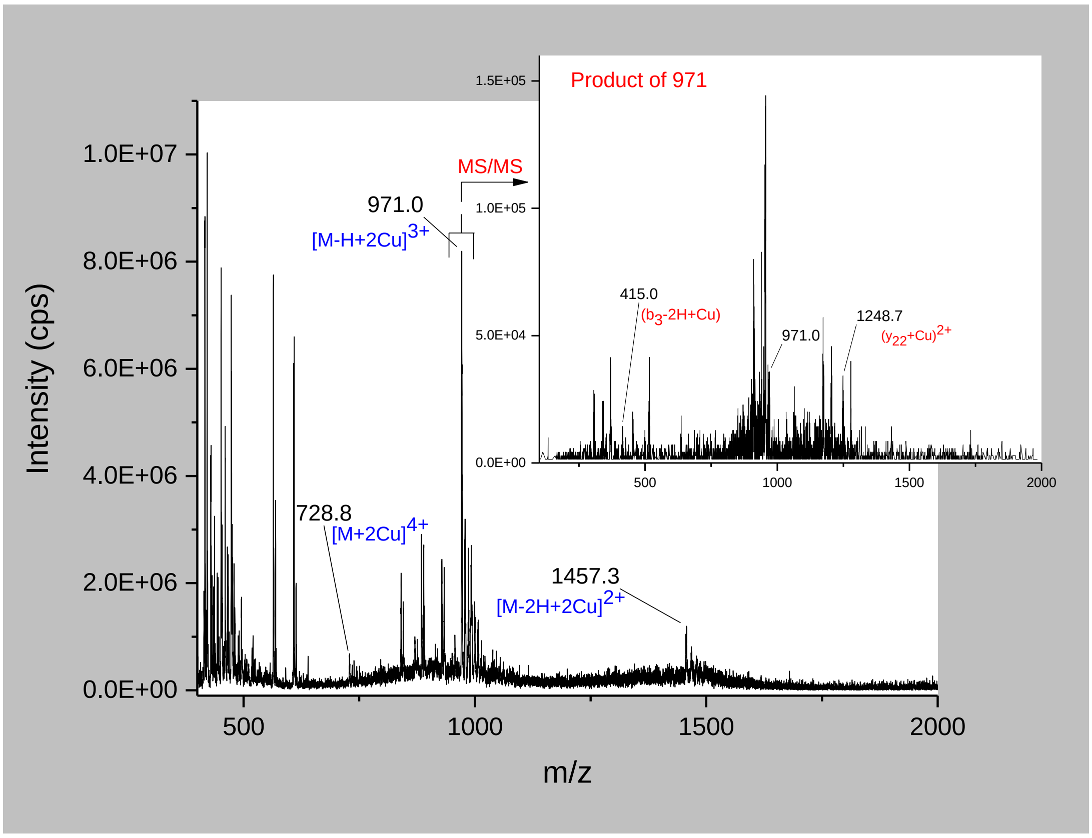

Fig. 5 – Full scan (MS) and product ion spectra (MS/MS) of hepcidin-25 complexed with two Cu(II) ions

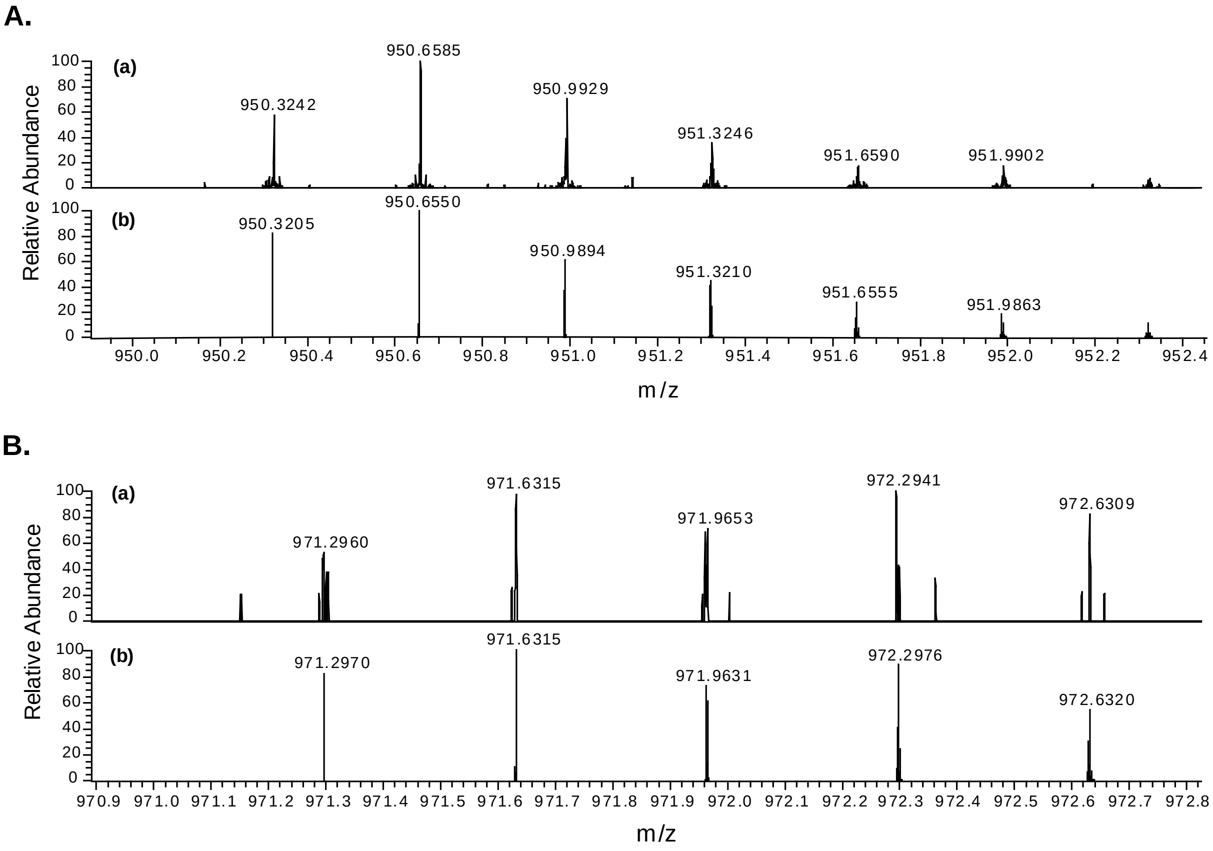

Fig. 6 – FTICR-MS spectra of the triply charged ion of hepcidin-25 complexed with A. one copper ion [M+H+Cu]$^{3+}$ and B. two copper ions [M+H+2Cu]$^{3+}$ (pH=11). The experimentally determined spectrum (a) is compared to the theoretical isotope pattern (b).

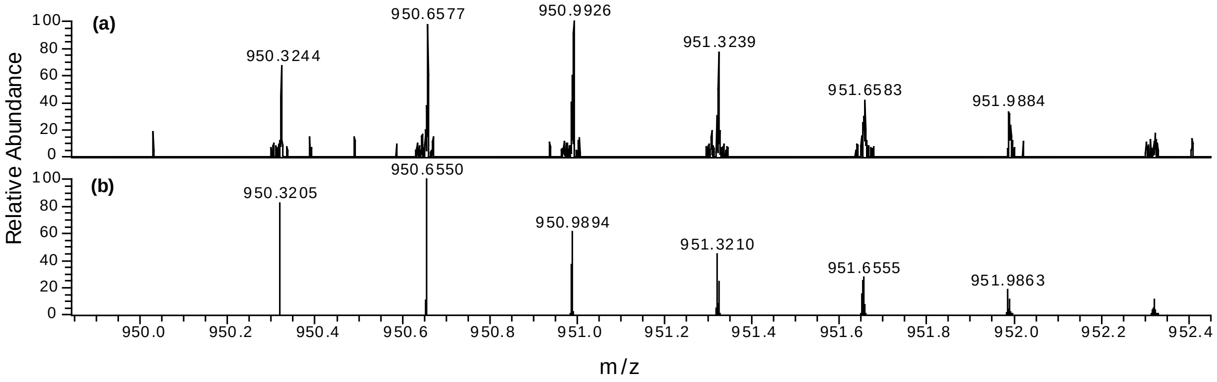

Fig. 7 - FTICR-MS spectra of the triply charged ion of hepcidin-25 complexed with one copper ion [M+H+Cu]$^{3+}$ (pH=7.4). The experimentally determined spectrum (a) is compared to the theoretical isotope pattern (b).

Hexapeptide-Ni^{2+} complex	
**NMR distances and dihedral constrains**	
Distance constraints	
Total NOE	36
Total dihedral angle restraints	6
**Structure statistics**	
Rms deviation	
Distance constraints (Å)	0.026±0.001
Dihedral angle constraints (◦)	4.328±0.064
Deviation from idealized geometry	
Bond lengths (Å)	0.028
Bond angles (◦)	3.3
RMSD - Backbone (Å)	0.63
**Ramachandran Plot Summary from Procheck**	
Most favoured regions	75.0%
Generously allowed regions	25.0%

Table 1 – Structure determination statistics of Ni^{2+}-bound hexapeptide (DTHFPI)

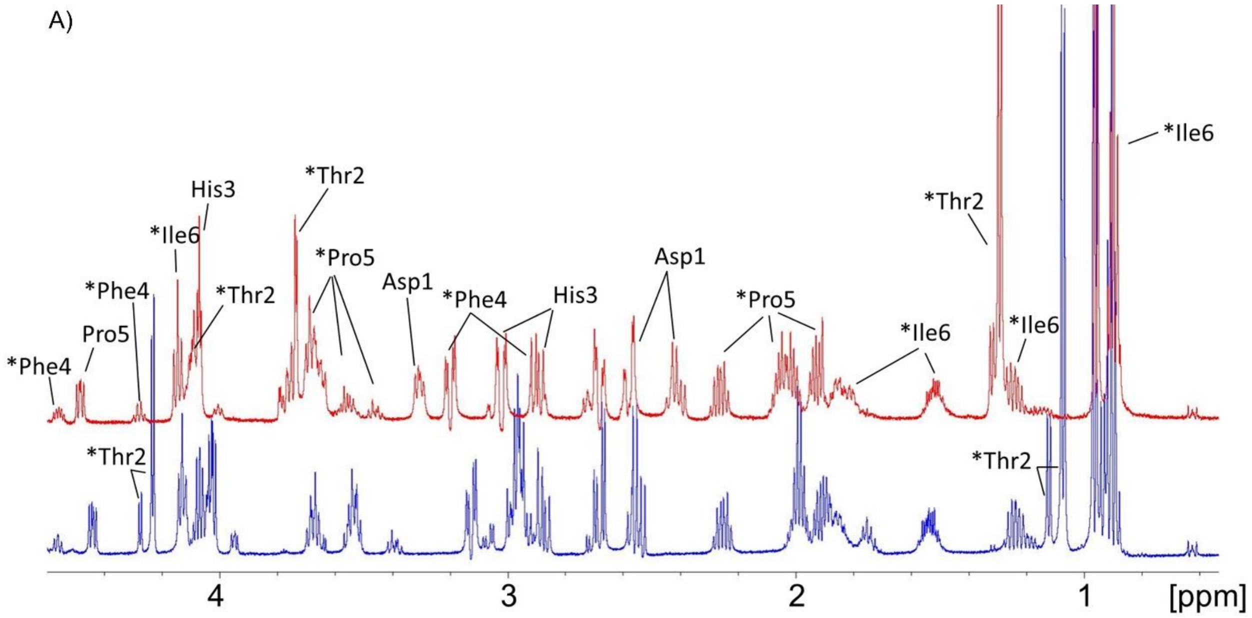

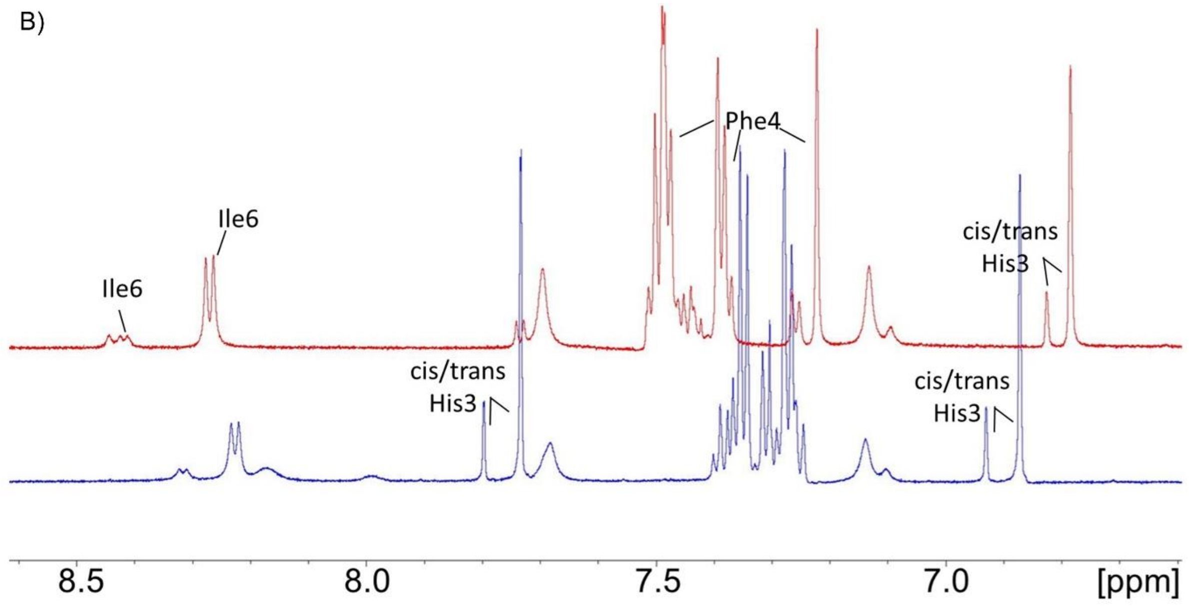

Fig. 8 – ***Metal-free and a metal-bound form of the peptide DTHFPI.*** *^{1}H NMR spectra of Ni^{2}-hexapeptide (1:1) complex (red) and uncomplexed peptide (blue). The largest chemical shift perturbation was observed for the N-terminal amino acids Asp-1, Thr-2, and His-3. Resonance assignment of A) the aliphatic and B) the aromatic region. * contains a double set of peaks for each amino acid and suggest a presence of cis-trans proline isomerization.*

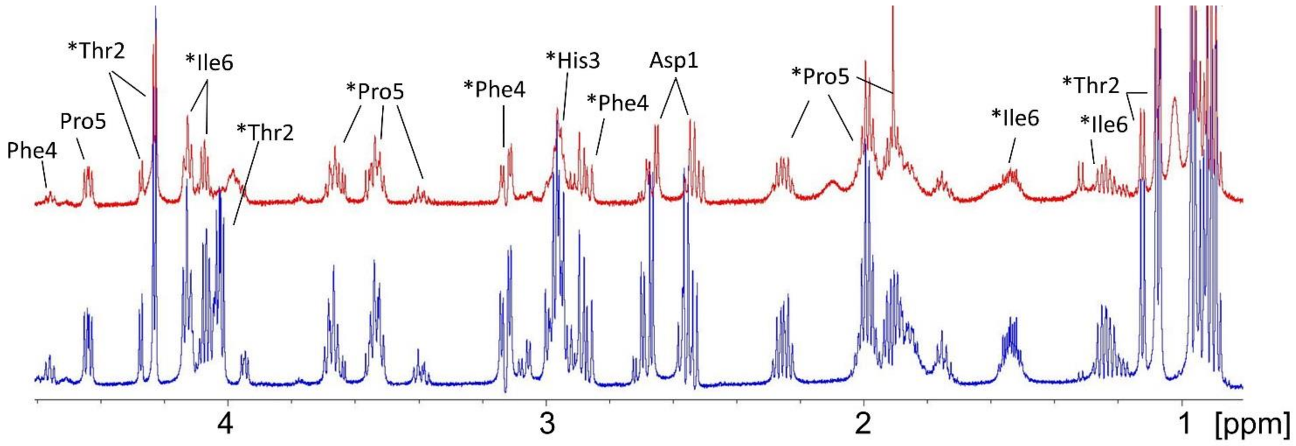

Fig. 9 – ***The copper-free and copper-bound forms of the DTHFPI peptide are in slow exchange.*** *^{1}H NMR spectra of the metal-hexapeptide complex in the presence of 0.5 copper(II) equivalent (red) and uncomplexed peptide (blue). The red spectrum confirms the slow copper(II) exchange between complexed and uncomplexed peptide. Due to the slow exchange, we were able to record the reduced signal intensity of the uncomplexed peptide even in the presence of the paramagnetic copper(II) complex. * contains a double set of peaks for each amino acid and suggest a presence of cis-trans proline isomerization.*

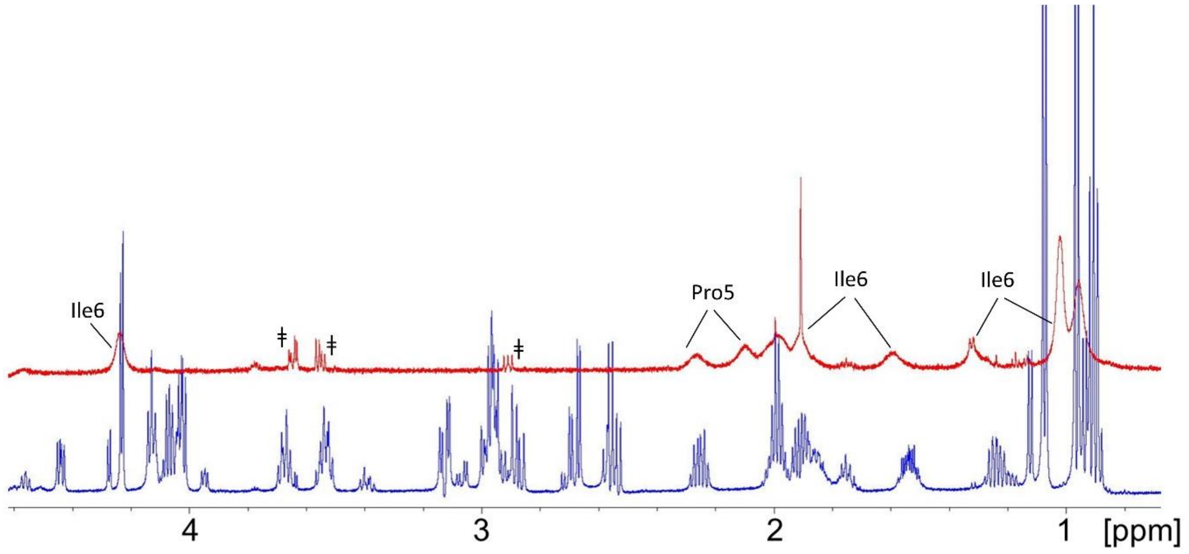

Fig. 10 – ***Paramagnetic line broadening in the copper(II) complex.*** *^1H NMR spectra of the Cu^{2+} metal-hexapeptide (1:1) complex (red) and uncomplexed peptide (blue). The paramagnetic effect of the copper (II) complex results in extreme line broadening of signals.* ***‡ Indicates the presence of the impurities.***

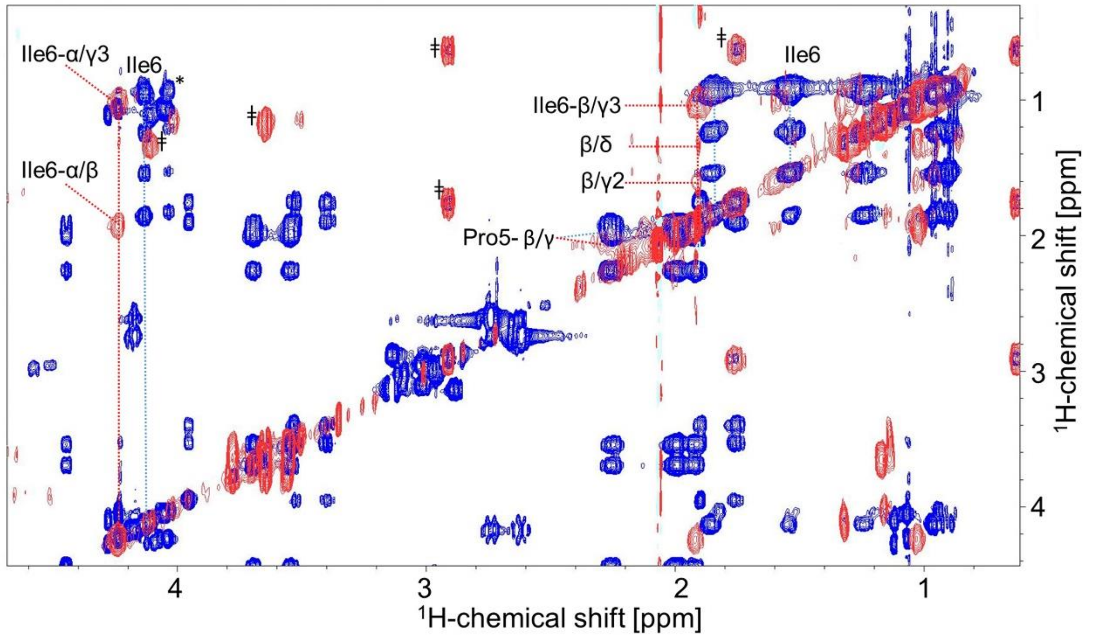

Fig. 11 – ***^1H-^1H TOCSY spectra of the paramagnetic copper complex.*** *DTHFPI hexapeptide in the presence of Cu^{2+} (1:1) complex (red) and in the absence of Cu^{2+} (blue). Resonance assignment of the hexapeptide-Cu^{2+} complex could only be obtained for amino acids with largest distance from the metal binding site, i.e. $H\alpha$ and sidechain protons of isoleucine [Ile-6] and sidechain protons ($H\beta$ and $H\gamma$) of proline [Pro-5]. ‡ Indicates the presence of the impurities.*

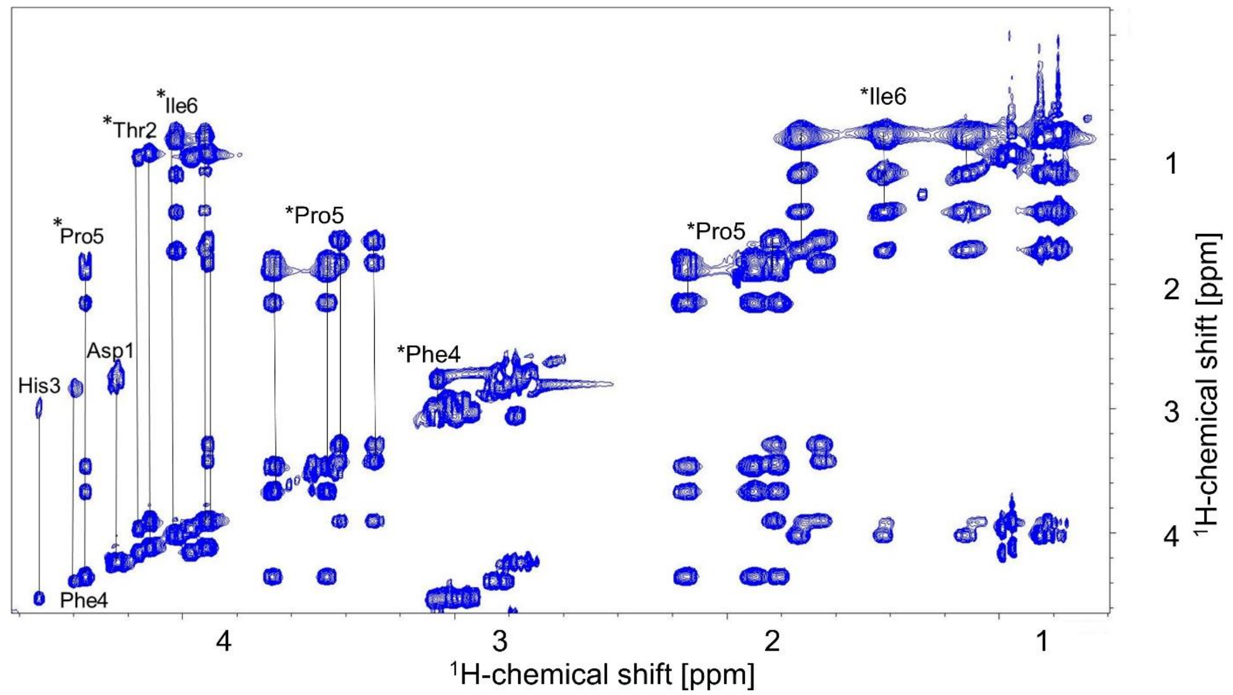

Fig. 12 – ***^{1}H-^{1}H TOCSY spectrum of hexapeptide DTHFPI.*** *The spectrum shows a 1 mM sample of hexapeptide recorded in 25 mM sodium phosphate, pH 3.2, 10% D_2O, T=298K. * contains a double set of peaks for each amino acid and suggest a presence of cis-trans proline isomerization.*

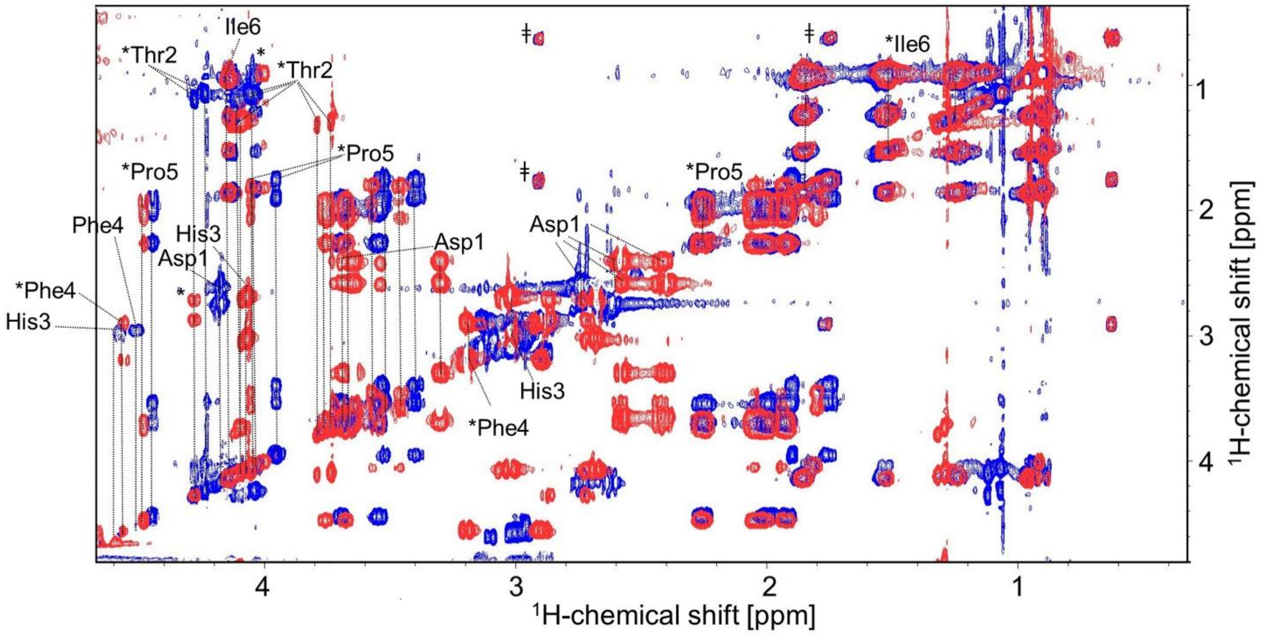

Fig. 13 – ***^{1}H-^{1}H TOCSY spectra of the hexapeptide-Ni^{2+} complex.*** *The resonance assignment of the hexapeptide in the presence of Ni^{2+} (1:1 complex, red) and in the absence of Ni^{2+} (blue).*
** contains a double set of peaks for each amino acid and indicative of proline cis-trans isomerization. ‡ Indicates the presence of the impurities.*

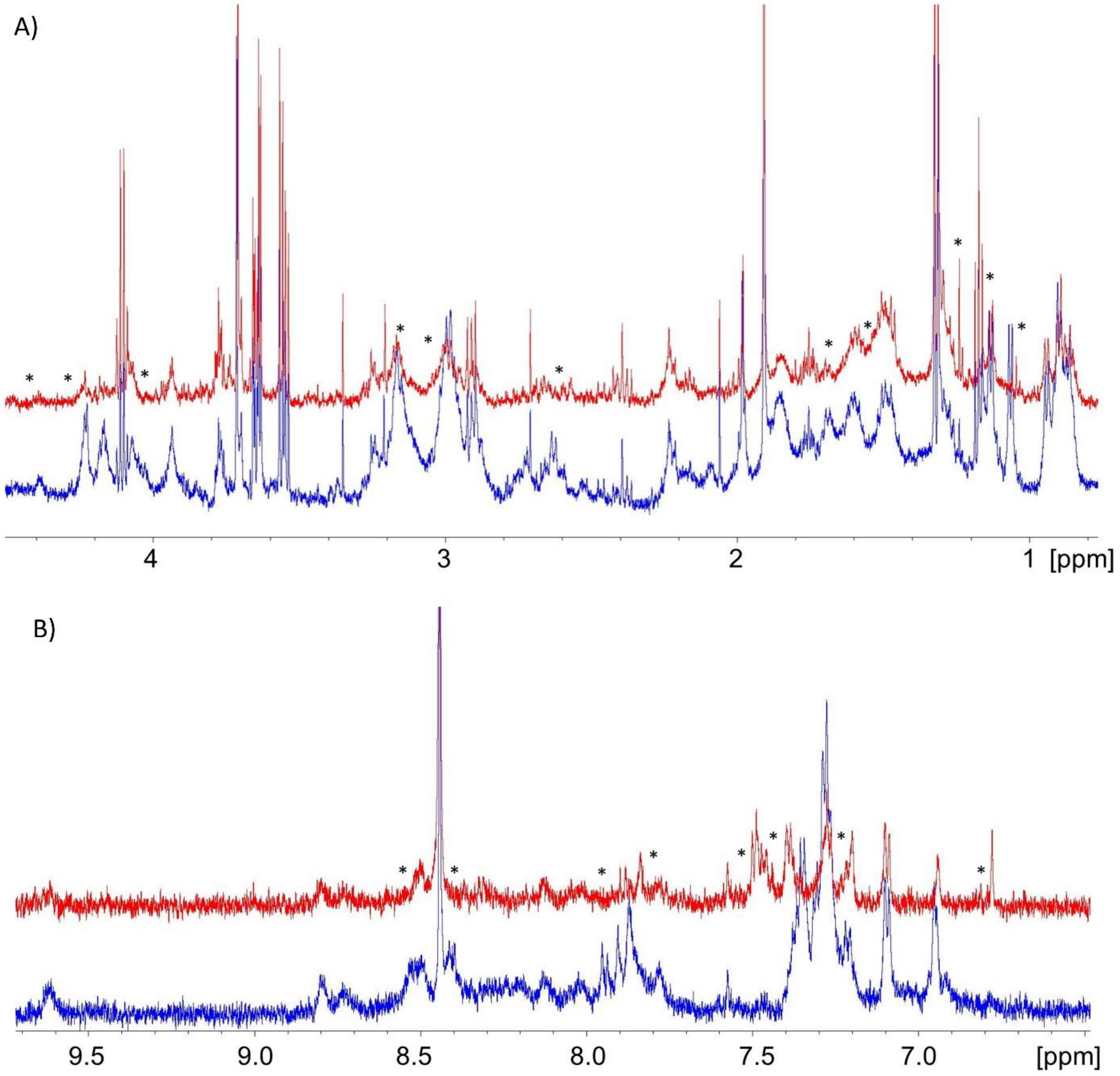

*Fig. 14 – **Metal-free and a metal-bound form of Hep-25.** ^{1}H NMR spectra of the metal-hepcidin 25 (1:1) complex (red) in the presence of Ni^{2+} and uncomplexed peptide (blue). The appearance of the new signals and the observed variation of the signal intensities clearly indicate that the two sets of signals correspond to a metal-free and a metal-bound form of the protein.*
Resonance assignment of A) the aliphatic and B) the aromatic region.
**changes in the intensity of peaks caused by metal binding*

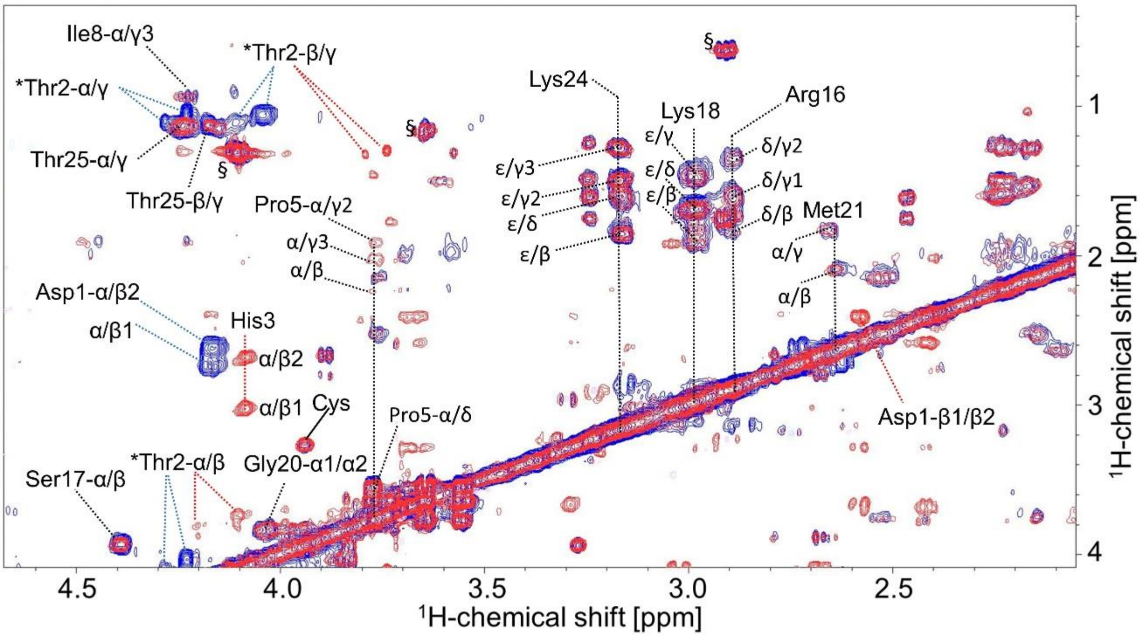

Fig. 15 – ***^{1}H-^{1}H TOCSY spectra of the hepcidin-25-Ni^{2+} complex.*** *The resonance assignment of hepcidin-25 in the presence of Ni^{2+} (1:1) complex (red) and in the absence of Ni^{2+} (blue).*
** contains a double set of peaks for each amino acid and suggest the presence of cis-trans proline isomerization. ǂ Indicates the presence of the impurities.*

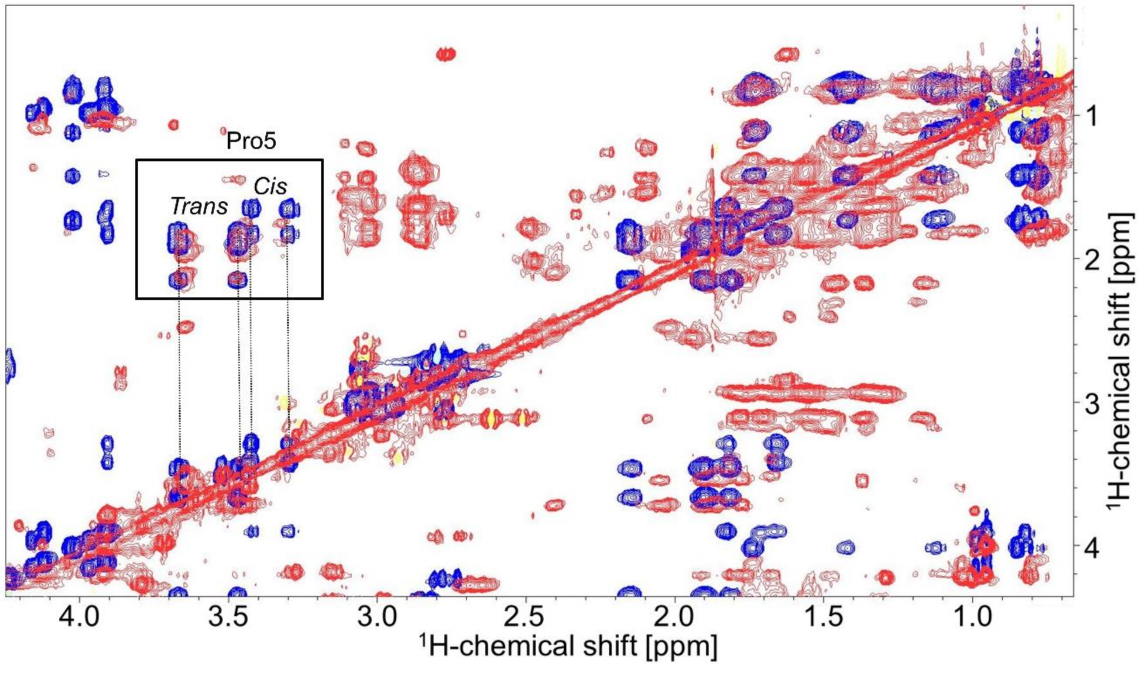

Fig. 16 - ***^{1}H-^{1}H TOCSY spectra of the hepcidin-25 (red) and hexapeptide (blue) at pH 3.***
We were able to distinguish the cis and trans proline isomers present in hepcidin-25 and hexapeptide in the same region of the TOCSY spectra.

The structural calculation of hexapeptide – nickel complex

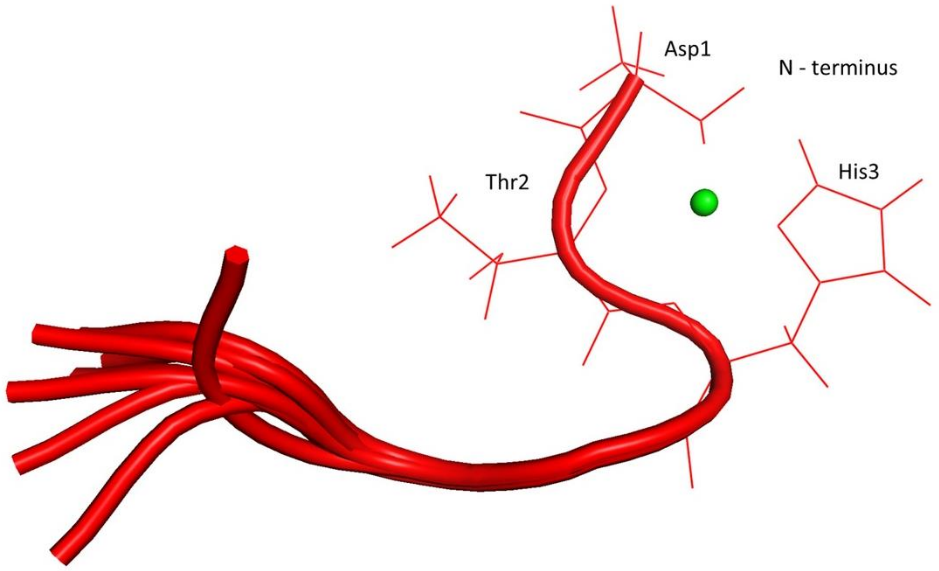

Fig. 17 - Superposition of ten refined structures of the Ni^{2+}-hexapeptide complex. The conformation of the three N-terminal, metal coordinating amino acids is very well defined by restraints imposing the square-planar coordination and by additional NOEs and dihedral angles. The two C-terminal amino acids show some structural heterogeneity. The available experimental constraints lead to a less well-defined structure compared to the N-terminus, and this part of the structure is likely to possess conformational flexibility.

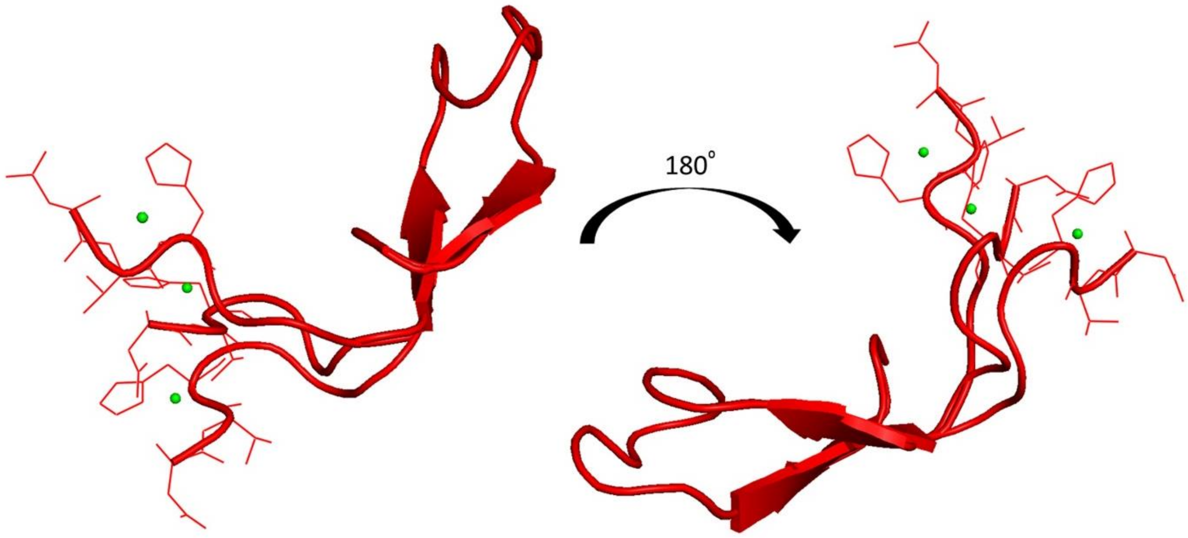

Fig. 18 - Superposition of three structures with lowest restraint violation energy of Cu^{2+}-bound hepcidin-25. As so far, no constraints stabilizing the arrangement of the ATCUN motif relative to the C-terminal part have been determined and because of the relatively low number of constraints for amino acids Phe-4, Pro-5 and Ile-6 found in the hexapeptide, the orientation and relative position of the metal center possesses significant flexibility in our simulations. It remains to be clarified if the N- and C-terminal parts share additional interactions that may stabilize one of these orientations.
